# Cell Mechanics Regulates the Dynamic Anisotropic Remodeling of Fibril Matrix at Large Scale

**DOI:** 10.34133/research.0270

**Published:** 2023-11-15

**Authors:** Mingxing Ouyang, Yanling Hu, Weihui Chen, Hui Li, Yingbo Ji, Linshuo Qiu, Linlin Zhu, Baohua Ji, Bing Bu, Linhong Deng

**Affiliations:** ^1^ Institute of Biomedical Engineering and Health Sciences, School of Medical and Health Engineering and School of Pharmacy, Changzhou University, Changzhou, 213164, China.; ^2^ Institute of Biomechanics and Applications, Department of Engineering Mechanics, Zhejiang University, Hangzhou, 310027, China.

## Abstract

Living tissues often have anisotropic and heterogeneous organizations, in which developmental processes are coordinated by cells and extracellular matrix modeling. Cells have the capability of modeling matrix in long distance; however, the biophysical mechanism is largely unknown. We investigated the dynamic remodeling of collagen I (COL) fibril matrix by cell contraction with designed patterns of cell clusters. By considering cell dynamic contractions, our molecular dynamics simulations predicted the anisotropic patterns of the observed COL bundling in experiments with various geometrical patterns without spatial limitation. The pattern of COL bundling was closely related to the dynamic remodeling of fibril under cell active contraction. We showed that cell cytoskeletal integrity (actin filaments and microtubules), actomyosin contractions, and endoplasmic reticulum calcium channels acting as force generations and transductions were essential for fiber bundling inductions, and membrane mechanosensory components integrin and Piezo played critical roles as well. This study revealed the underlying mechanisms of the cell mechanics-induced matrix remodeling in large scales and the associated cellular mechanism and should provide important guidelines for tissue engineering in potential biomedical applications.

## Introduction

Tissues and organs in the body often have anisotropic structures or shapes, in which developmental process is coordinated by cells and extracellular matrix (ECM) modeling [1,2]. Abnormal ECM remodeling is often thought as a sign of disorders, such as fibrosis in liver and lung, cartilage degradation in joints, and ECM-associated genetic diseases [3]. Several studies demonstrated that tumor cells are able to reconstruct ECM environments in facilitating the metastasis [4–6]. Cells also show the capability in remote ECM modeling, for example, 2 explanted fibroblast tissues could build fiber bundles across 1.5- to 4-cm distance on the collagen hydrogel [7,8]. Hence, cells can format specific ECM structure that in return mediates the physiological outcomes of cells and tissues. Besides the chemical cues, the biomechanical aspect of how cells and ECM coordinate at the large spatial scale during morphogenesis and for tissue engineering has been an interesting topic to explore.

Cell mechanical communications are recognized from in vitro and in vivo studies [9,10]. Different from traditional chemical cues, mechanical communications between cells show the features with long-range or large-spatial-scale transmission, precise direction, and functional outcomes at the tissue scale. For example, cells are able to sense the positions of neighboring cells through compliant substrates [11,12]. Long-range force enables assembly of mammary tissue patterns and collagen fibrillary remodeling [13,14]. Cardiomyocytes achieved similar beating frequency when seeding on elastic substrate [15,16]. During *Xenopus* embryonic development, the cell cluster of neuronal crest moves collectively to the destination with supracellular contraction at the rear to assist directional migration [17,18]. Invasive breast cancer cells showed collective migration resulting from traction force transmission through Matrigel hydrogel [19]. These increasing evidences have supported the long-distant mechanical communication as a common nature of cells.

The cellular mechanism on mechanical communications has also been studied. As shown from recent work including ours, cells have the capability to sense the traction force or substrate deformation transmitted from neighboring cells or induced by external probe, which provides a bridge in understanding cell–cell distance interactions [20–24]. At the molecular level, the mechanosensitive receptor integrin and ion channel Piezo display importance in remote attractions between 2 types of cells [25,26]. The calcium channels on endoplasmic reticulum (ER) are also indispensable for cell remote mechanical interactions [27]. There is also long-distance force transmission through cell–cell junctions during the collective cell migration [28,29]. So far, although some mechanisms and signals have been identified for the mechanical communications, the general picture at this aspect is still unclear.

Interestingly, cells are found to be able to reconstruct the collagen I (COL) gel to generate fibrous bundles [8,30], of which the biophysical mechanism has attracted research attentions. It was shown the active physical force from tissues able to rearrange COL patterns with long-range order [31]. A later study confirmed that the local movements of COL was driven by fibroblast contractions leading to the fibrillary organization [32]. In the recent decade, more research works employing mechanical approach have emerged on the cells-driven fibrous morphogenesis. For instance, the finite element (FE) simulations were adopted to predict the alignment of COL gel induced by long-range biomechanical transmission, although the results were conditional with a pair of polarized cells [33,34]. Continuum modeling with FE simulations further recapitulated the measurements of cell traction force propagation [35]. Application of optical tweezers to pull beads on the COL hydrogel found the long-range stiffness gradients surrounding the cell [36]. In the synthetic fibrous materials with controllable biomechanical architectures, cell contraction induces the alignment of fibers [37,38]. By mimicking in vivo condition, experimental stretch or fluidic shear stress were also able to align long-range order of ECM [39,40]. A recent study showed that cells-reorganized fiber bundles bear the major tensile force to guide cell distant interaction and correlated migration [41]. In general, the problem of how to understand the cells-induced ECM remodeling at large spatial scale is still challenging.

Apparently, intracellular and intercellular signaling plays crucial roles in cells’ remodeling ECM in various physiological and pathological conditions. For example, cancer-associated fibroblasts can induce COL cross-link switch and align fibrous COL or fibronectin to promote fibroblast long-distance order or direct cancer cell migration [4,6,42]. Early-stage cancer cell spheroids embedded into COL containing fibroblasts generated force for fibers alignment and cancer-associated fibroblast induction [43]. Our work also showed that motile kidney epithelial cells can actively recruit soluble ECM from the medium to assemble base membrane or COL architectures in directing spherical or tubular tissue formations [44,45]. The cell signals mediated by integrin, RhoA, and actomyosin contraction show pivotal roles in cells-regulated ECM remodeling [4,46]. However, the fundamental molecular mechanisms in the remote ECM modeling is largely unknown.

In this work, we aim to study the long-range COL fibril remodeling induced by cell active contraction force, and the associated mechanosignaling mechanisms in cells. We considered a large-scale array of cell clusters that perform dynamic contraction in the matrix. This research design eliminated the limitations of previous conditional studies, such as those adopting single or a few cells/clusters with polarized cell shape, or static traction force [47]. In our previous works, we had shown that the traction force of live cells is dynamic instead of static in the hydrogel matrix, induced by the highly motile cell clusters with constant rotation [13,44], as reported by the Tanner and Bissel groups as well [48]. Here, by employing the molecular dynamics (MD) simulation that incorporates dynamic traction of cells, we recapitulated the experimental observation of COL fiber bundle formation without the conditional spatial-scale limitation. The important roles of different cellular mechanical components were further verified in the COL bundling inductions, such as force generation (actomyosin), transduction (calcium signals, cytoskeletons), and mechanosensors (integrins, Piezo).

## Results

### Cell-mechanics-induced large-scale COL fibrillary remodeling for different patterns of cell clusters

**Fig. 1.**
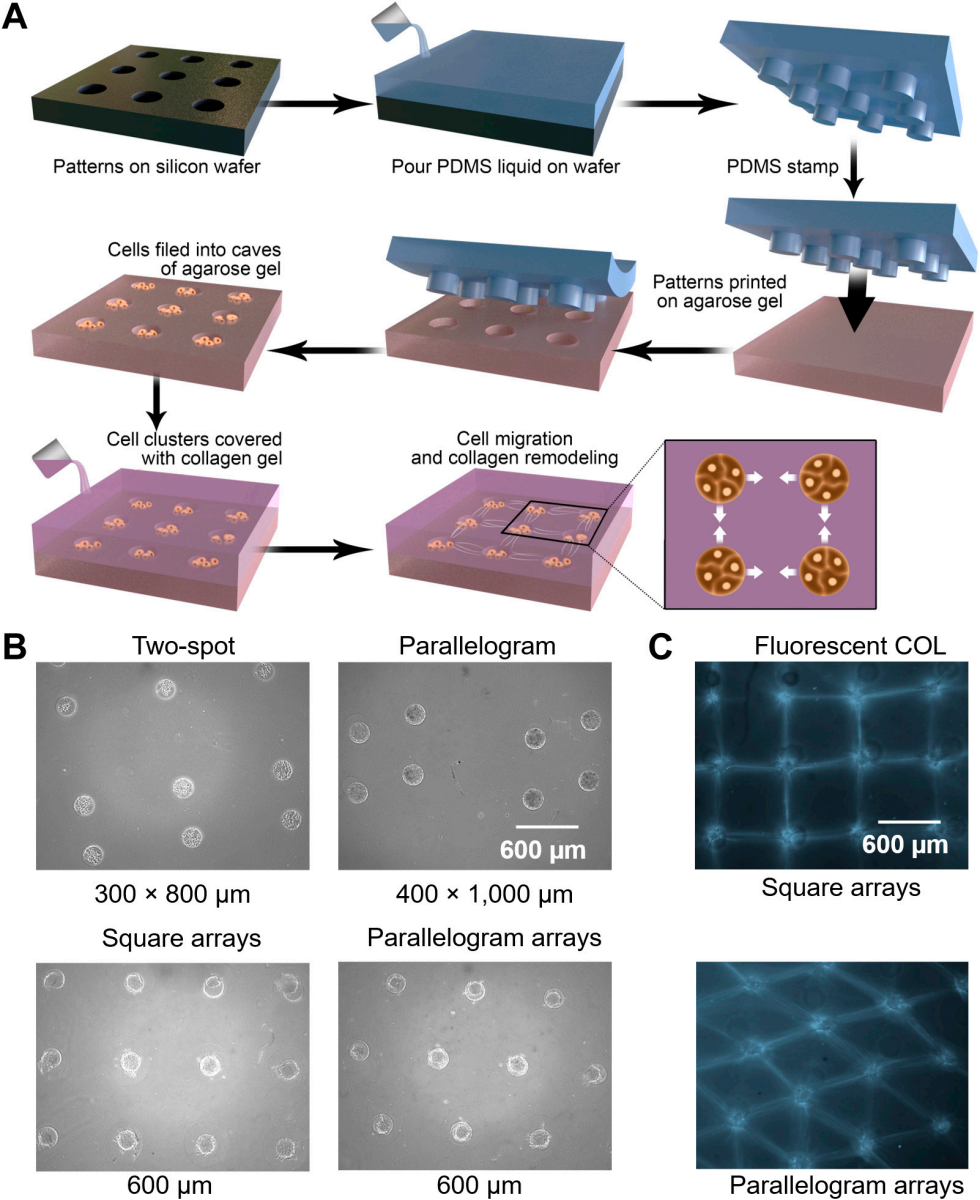
Experimental observation of COL gel remodeling under the contraction of cell clusters of various patterns. (A) The diagram of experimental procedures described in Methods. (B) The representative images of cell clusters positioned in different geometries. Distance between clusters is shown under the images. (C) The emerging COL fiber bundles in square and parallelogram arrays after 48-h cultures.

To explore the mechanism and robustness of the COL gel remodeling induced by cell contraction, we designed various micropatterned arrays of cell clusters according to the procedures from our previous works [13]. The diagram in Fig. 1A shows the experimental steps in preparation, of which the details are described in Methods. Representative images of cell clusters arranged into different geometries are demonstrated in Fig. 1B, including a single pair, single parallelograms, square arrays, and parallelogram arrays of cell clusters. More geometrical patterns will be shown later in the following figures. The single-pair results were recently published in a journal in Chinese [49]. The networks of COL fiber bundles were emerging in the square and parallelogram arrays as shown in Fig. 1C.

### MD and FE simulations of COL fiber remodeling driven by cell contraction induced stress field

**Fig. 2.**
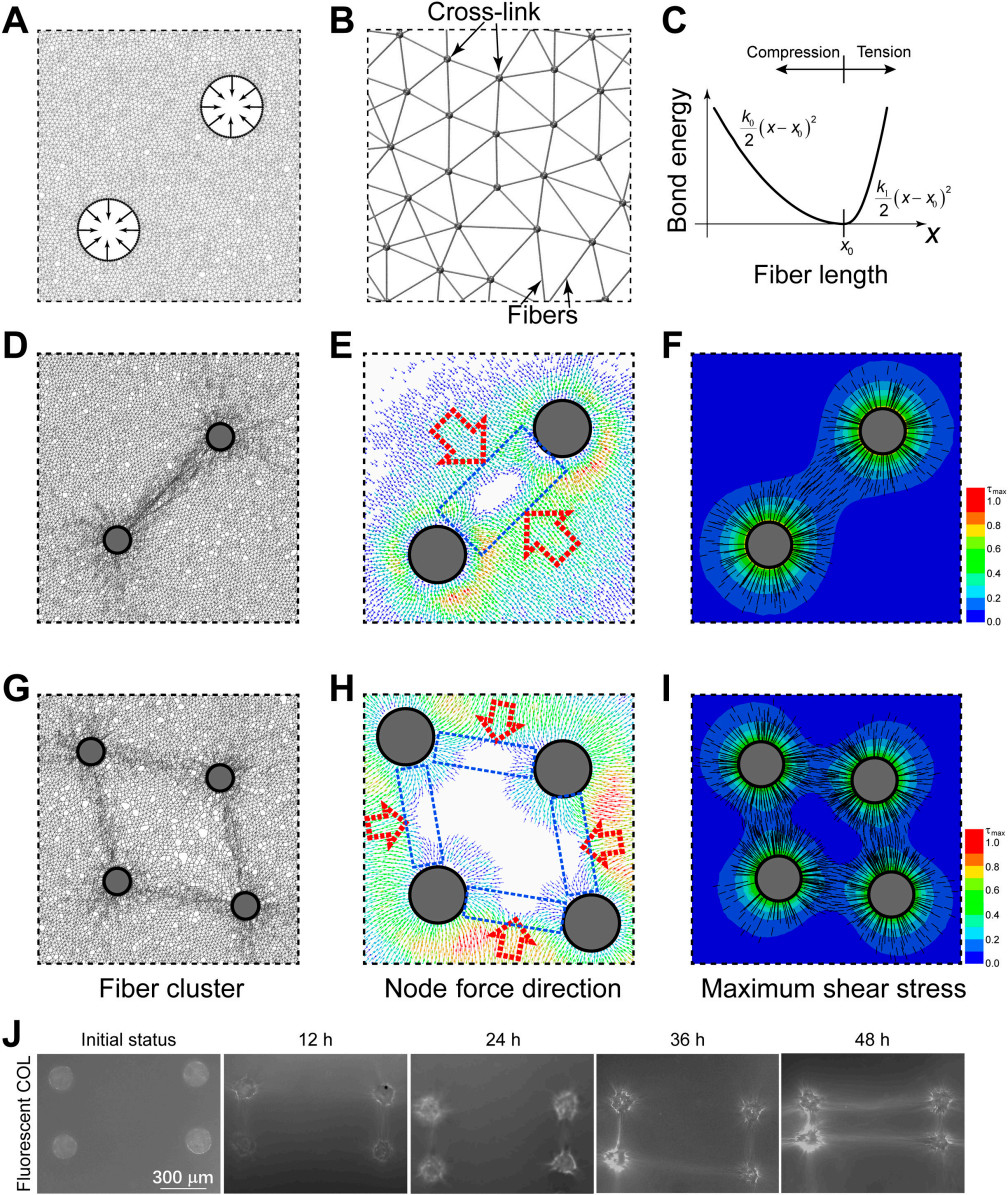
The relationship between the fiber bundling and the cell active stress field. (A) The MD model for simulating fiber bundling. The contraction force by cells was applied on the interface between cell and fibril matrix. (B) A zoom-in view of the fibers bonds network. (C) The bond energy changes with the bond length. The tensile stiffness of the bond is higher than the compressive stiffness. (D) Fibers were remodeled along the line connecting the centers of 2 cell clusters. (E) The particle/node force distribution and the direction of particle/node forces in the case of 2 cell clusters predicted by MD simulation. (F) The maximum shearing stress field and the direction of the first principal stress in the case of 2 cell clusters. (G) Fibers were remodeled in the line connecting the centers of 2 neighboring cell pairs in the condition of 4 cells. (H) The node force distribution and the directions of particle/node force for the 4-cell cases predicted by MD simulation. The colors from blue to red represent lower to higher simulated stress in (E) and (H). (I) The maximum shearing stress distribution on matrix and the directions of first principle stress in the case of 4 cell clusters. (J) The representative experimental result of fluorescent images of COL fiber bundling induced by cell clusters at the given culture time. The center distances of the paired clusters and between 2 pairs are 500 and 1,000 μm, respectively.

To study how the cell traction force remotely drives the formation of COL fiber bundles in the hydrogel, we built a 2-dimensional (2D) network model based on MD simulation method to investigate the direction and mechanics of fibers remodeling (Fig. 2A). The collagen fibers were modeled by 2D fibers consisting series of bonds/beads, and their cross-links were modeled as their jointed nodes between/among different fibers (Fig. 2B). The collagen fiber is modeled as an anisotropic elastic material that has a high modulus in tension and a very low modulus in compression (Fig. 2C) [50].

To investigate how the stress field regulates the fiber remodeling, the force direction on each node and the maximum shearing stress on matrix was calculated by molecular dynamic simulation and finite element modeling (FEM), respectively. MD simulation predicted that under the dynamic contraction of cells, the fibers remodel and form bundle-like structure between 2 neighboring cells (Fig. 2D). The particle force exhibited similar pattern of distribution (Fig. 2E). Considering the orientation of node force, the fibers were bearing a pulling force along the connection line between cells, which would lead to aggregation of the fibers (seen in Fig. 2D and E). The local maximum shearing stress distribution on matrix was calculated as shown in Fig. 2F, and the black line revealed the direction of the maximum principle stress. The maximum shearing stress has a relatively high value around the connection line of 2 neighboring cells, and this region is just where the fibers aggregated (Fig. 2F).

For the condition of 4 cells, fiber aggregation formed in closer cell pairs instead of those with larger distance (seen in Fig. 2G). Similar to the results of 2 cells, the fiber lines are also in agreement with the first principle stress direction and the particle force distributions (Fig. 2H). Although the central region has higher first principle stress, the maximum shearing stress is lower than the connecting lines of cell pairs (Fig. 2I). Experiments with seeded cell cluster pairs demonstrated the COL fibers emerging between clusters (Fig. 2J), consistent with the MD-simulated outcomes. Taken together, fibers were remodeled in the region with higher maximum shearing stress along the orientation of the local maximum principle stress.

### Large-scale COL fiber remodeling induced by cell clusters in square and parallelogram-like patterns

**Fig. 3.**
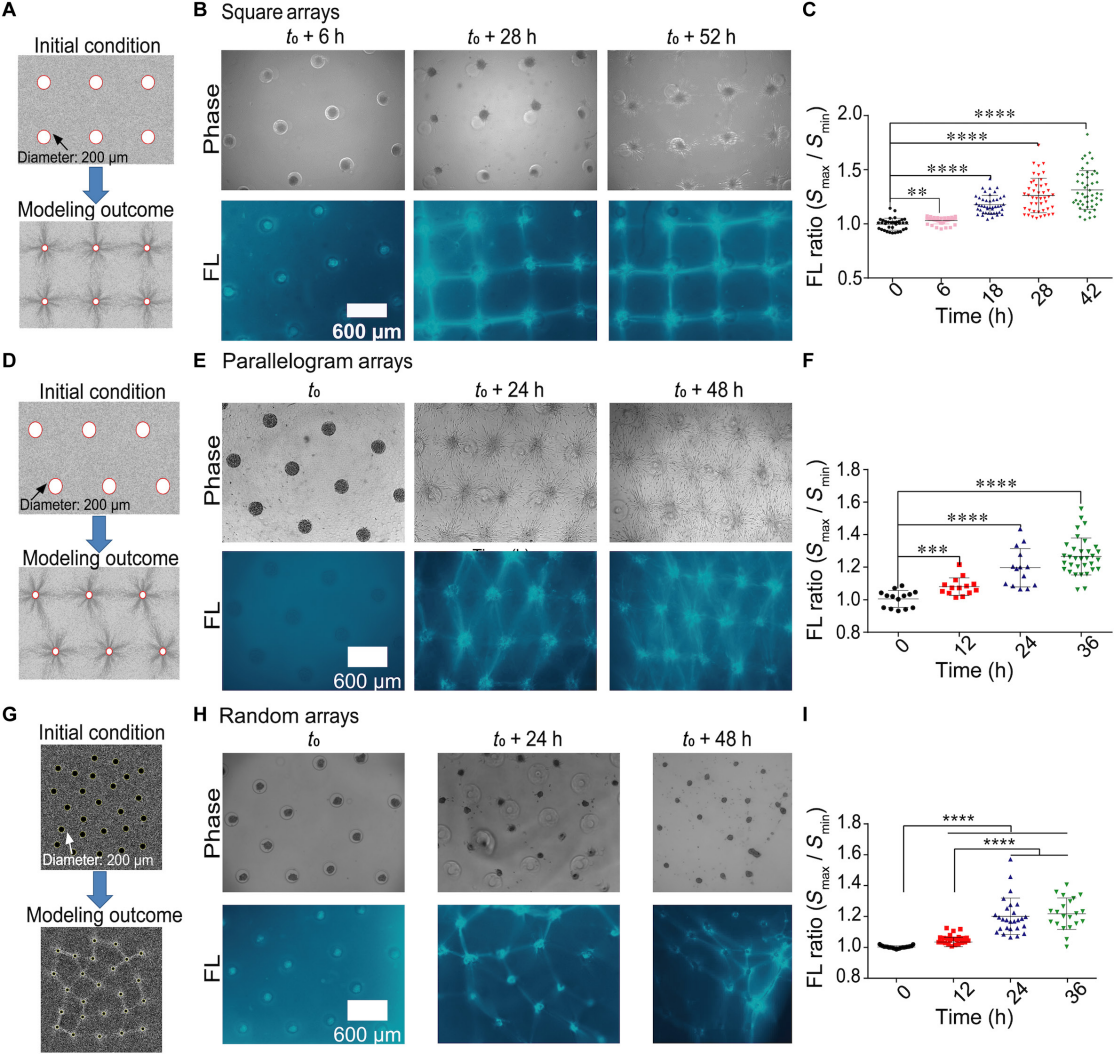
COL fiber bundling in various patterns of cell clusters at large scale. ASM cell clusters were seeded into the designed geometrical arrays with fluorescent COL hydrogel (1 mg/ml) atop. Images of cells and COL fluorescence were taken at the indicated time. (A) MD simulation results of COL fiber bundling in square pattern. (B) The experimental results of COL fiber bundling in the square pattern. (C) Fluorescence (FL) quantification of COL fibers (mean ± standard error of the mean (SEM.) in contrast to the nonfiber region based on the ratio calibrations (*S*_*max*_/*S*_*min*_). Each dot represents the ratio value of one fiber bundle. (D to F) The MD simulation results (D), the emerging COL fiber bundles (E), FL ratio quantifications (F), and fluorescence distributions (H) from parallelogram-arranged cell cluster pattern. (G to I) The MD simulation outcome (G), the emerging COL fiber bundles (H), and FL ratio quantification (I) from the geometrical random-style cluster pattern. The statistical significance for COL fluorescence between the initial and indicated time points was evaluated by Student *t* test. *, **, ***, **** represent *P* value < 0.05, 0.01, 0.001, 0.0001, respectively, and so on through the paper.

The simulation results of MD showed that fiber bundling appeared mostly at the edges of pattern by the dynamic contraction of cell cluster arrays in a square pattern (Fig. 3A). The experimental results were obtained by seeding cell clusters into square or parallelogram patterns covered with fluorescent COL gel (1 mg/ml, labeled with EGFP-CNA35), and images were taken at specific time points in the next 2 d. The data showed the emerging square lattices of COL fiber bundles, connecting of the clusters in the pattern (Fig. 3B). More details of the fiber growth processes are shown in Supplementary Materials (Fig. S2A). The fluorescence quantification (Ratio = *S*_*max*_/*S*_*min*_) and intensity distributions indicated the gradual growth of the fibers (Fig. 3C and D). The ratio started at ~1.0 as an initial uniform fluorescence distribution and reached ~1.2 to 1.5 as fiber growth between cell clusters. Fluorescence intensity curves of individual selected region of interest (ROI) regions are presented in Fig. S3. The lattice-like fiber bundle structure stayed stable in the 2-d observations, indicating balanced contraction force through the square array.

We next examined the results in parallelogram pattern. MD simulation showed that fiber bundles formed mostly at the edges of the pattern while there were less at the diagonal directions (Fig. 3E). In the experiment, when cell clusters were seeded into the parallelogram pattern, COL fiber buckling occurred mostly at the sides, resulting into formation of parallelogram lattices (Fig. 3F, more details shown in Fig. S2B). Fluorescence quantification and spatial distributions indicated the gradual accumulations of COL fibers between cell clusters (Fig. 3G and H and Fig. S3B). As seen at later time (e.g., 36 h) (Fig. S2A and B), some fiber bundles showed up at the diagonal directions in parallelogram and square arrays. This experimental difference from the simulations might be partially due to the changed material property under remodeling by the cell clusters, or the simulations could not completely mimic what was going on in the experiments yet, of which the explanation needs further study.

We further applied random-style patterns of cell clusters (mutual spatial separations within 600 to 1,000 μm) to check the simulation and experimental outcomes for fiber growth. The MD simulation well turned-out fiber emergences in connecting cell clusters (Fig. 3I), the dynamic process of which is shown in Movie S1. Experiments based on random-positioned arrays showed gradual fiber growth, resulting into fiber network formation along with clusters (less stable structure) (Fig. 3J and K and Fig. S2C), which indicates the robustness of cell pattern-induced fiber assembly. Hypothetically, the fiber bundling in the random array was driven by cell maximal stress distributions. Comparison of the COL fiber bundle strengths indicates that the square arrays had stronger induction of fibril assembly than the parallelogram and random ones (Fig. S2D). Therefore, the experimental results of fiber bundling within various geometries due to the dynamic tractions of cells at the large scale were well consistent with MD/FEM simulation results. This MD and FEM combined simulation demonstrated robustness in predicting the experimental outcomes without cell shape, culture boundary, or spatial-scale limitations in comparison to previous studies [34,47,51,52].

### COL fiber assembly in the single polygon-like pattern with varied geometries

We further designed patterns of cell clusters in single polygon-like style with varied geometries including triangles, parallelograms, squares, pentagons, and hexagons (600 μm in mutual separations of the centers). This design allows us to assess the robustness of cell contraction-induced fiber assembly and the stability of assembled fiber structures.

**Fig. 4.**
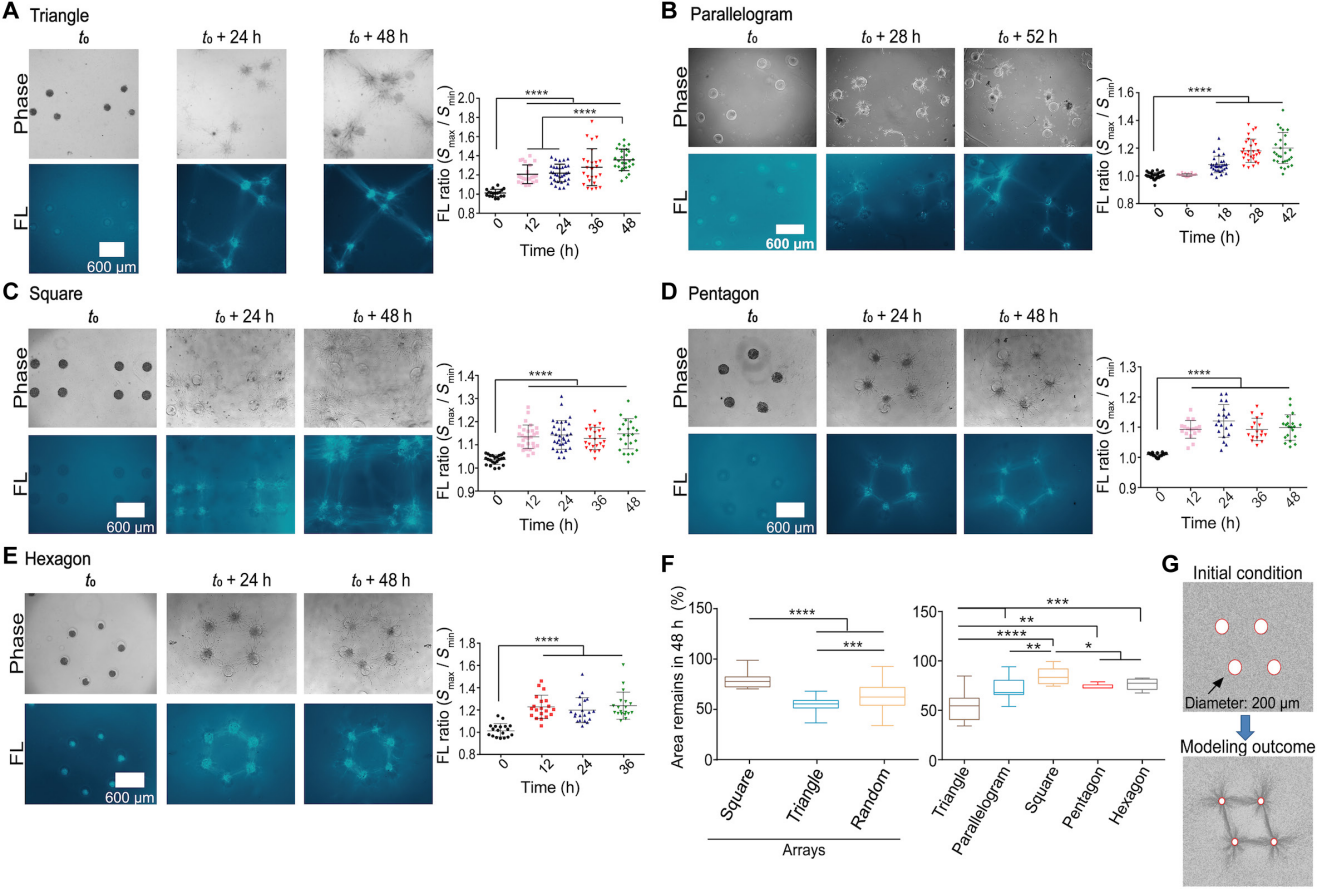
Fiber assembly in single polygon-like pattern with varied geometries and their structural stabilities. Cell clusters were seeded into single polygons. Images were acquired at different time points, and fluorescent COL fibers were measured as Ratio = *S*_*max*_/*S*_*min*_. (A to E) The growth of COL fibers and fluorescence quantification (mean ± SEM) for fiber strength in single triangles (A), parallelograms (B), squares (C), pentagons (D), and hexagons (E). (F) Stability measurements with remained areas (%) from geometrical shrinking for the diverse arrays (from Fig. 2) and single polygons within 48 h. The mean ± SEM values of remaining area (%) for square array: 79.5 ± 1.3; parallelogram array: 55.3 ± 0.7; random array: 63.3 ± 1.8; single triangle: 54.8 ± 3.5; parallelogram: 71.6 ± 2.6; square: 82.4 ± 2.4; pentagon: 74.3 ±1.2; hexagon: 75.2 ± 1.9. (G) The MD simulation for fiber clustering in a single parallelogram.

When cell clusters were settled down into single triangles, strong COL fibers were shown up at the 3 sides rapidly (within 12 h), and cells migrated toward each other (Fig. 4A, left). There was also fiber growth between 2 triangles which had 1,000 μm in distance. Group fluorescence quantification confirmed fiber assembly in the designed triangles (Fig. 4A, right). In single parallelograms, COL fibers emerged gradually at the 4 sides along with some shown up at the diagonal lines (600 μm in distance) (Fig. 4B). In single squares, COL fibers occurred at the sides, not much in the diagonal direction (848 μm in distance) (Fig. 4C). When cell clusters were arranged into single pentagons or hexagons, COL fibers were shown up efficiently at the sides but not at the diagonal lines (Fig. 4D and E), the distances of which between cell clusters at the diagonal directions (~1,141 and 1,039 μm in pentagons or 1,200 μm in hexagons) were much larger that at the sides (600 μm). Cells also migrated toward each other along the sides.

By measuring the area changes within 48-h culture to evaluate the structural stabilities shown in Fig. 3, the square arrays were more stable than parallelogram and random-style ones (Fig. 3F, left). The single polygons (Fig. 4A to E) were quite stable (less than 30% shrinking in areas), except triangle ones (~45% shrinking) (Fig. 3F, right). More details for fiber inductions from single triangles to hexagons are presented in Fig. S4A to E. As a demonstration, MD simulation for single parallelogram predicted fiber clustering mostly at the sides (Fig. 4G) and similar to FEM simulation of the maximum stress distribution (Fig. S4F). Comparison of COL fluorescence ratio among different single polygons shows stronger fibril inductions in triangles, parallelograms, and hexagon than in squares and pentagons (Fig. S4G). The underlying mechanism for this difference has not been identified yet in this work. Together, these results from above show the recapitulation of dynamic simulations for experimental outcomes (especially at early time stages) and demonstrated fiber assembly induced by cell remote mechanics in COL hydrogel.

### The cytoskeleton integrity and actomyosin contraction are essential for the formation of COL fiber bundles

**Fig. 5.**
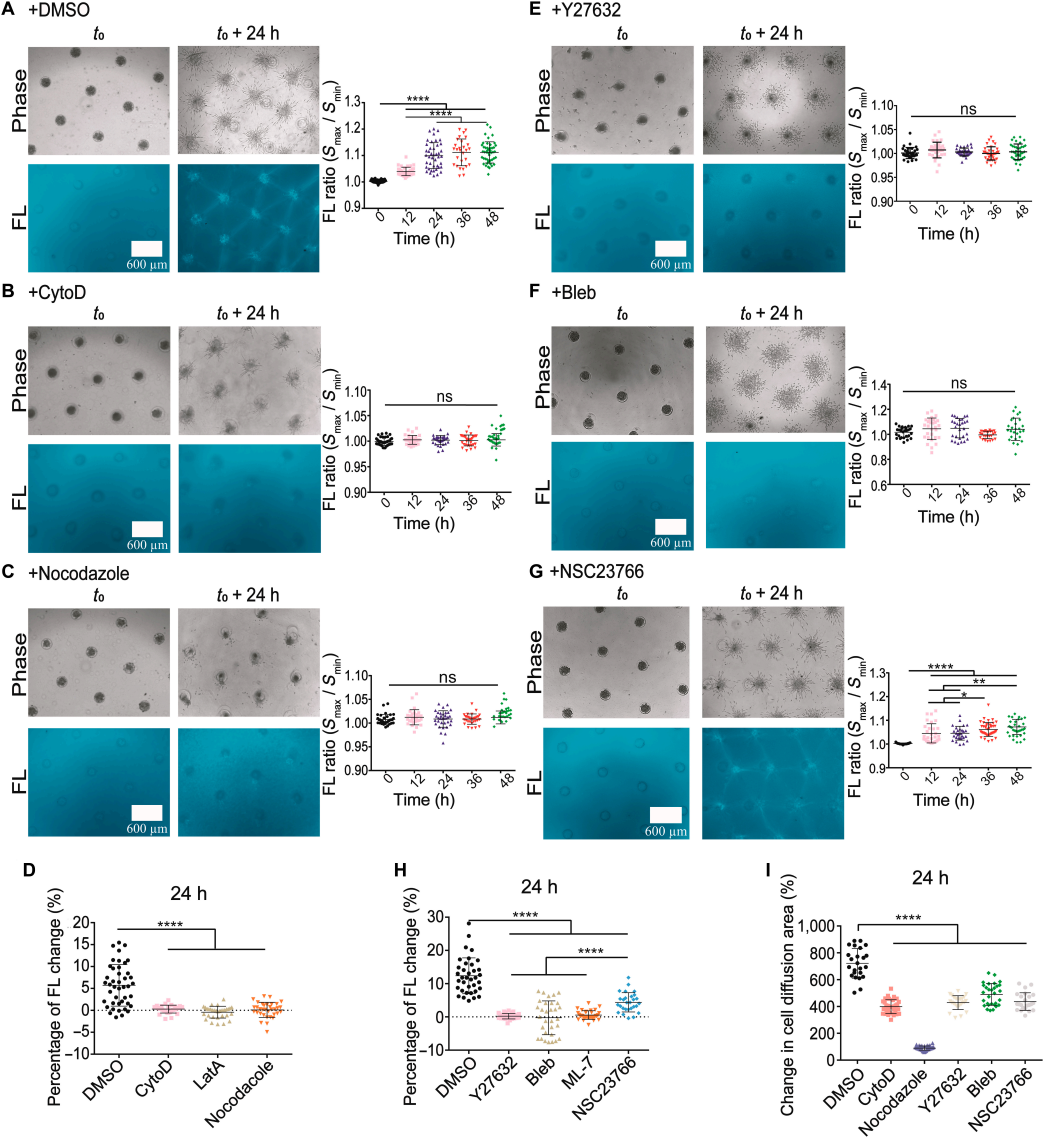
Cytoskeleton integrity and actomyosin contraction in remote induction of fiber assembly. Cell clusters were cultured into large parallelogram pattern with cytoskeleton or actomyosin inhibitors. (A to C) The COL fiber growth and fluorescence quantification at control condition (DMSO) (A), or under treatment with 1 μM CytoD (B), or 1 μM nocodazole (C). (D) Comparison for the percentage changes of COL fiber fluorescence within 24 h with or without cytoskeleton inhibitors. (E to G) The COL fiber growth and fluorescence quantifications under treatment with 40 μM Y27632 (E), 40 μM blebbistatin (F), or 40 μM NSC23766 (G). (H and I) Comparison for fiber fluorescence change (percentage) (H) or cell spreading areas from the clusters (I) within 24 h at the indicated conditions. The experimental data with LatA or ML-7 treatment were shown in Fig. S6.

Since cells induced fiber assembly in the experimental models, we further looked at the involved cellular biomechanical mechanism by employing large-spatial parallelogram arrays. First by checking the importance of cell cytoskeletons, cells actively migrated out from clusters toward each other and developed COL fiber networks at control condition (0.1% dimethyl sulfoxide [DMSO]) (Fig. 5A); when actin cytoskeleton was inhibited with cytochalasin D (CytoD) or latrunculin A (LatA) treatment, the fiber assembly was gone, and cells did not migrate out actively (Fig. 5B and Fig. S5C); similar results were observed when microtubule cytoskeleton was inhibited with nocodazole (Fig. 5C). The comparison of percentage changes in COL fluorescence quantification showed inhibited fiber assembly by the 3 drug treatments (Fig. 5D). The representative images with more details for cytoskeleton inhibitions are presented in Fig. S5A to F. These data indicate that the integrity of both cellular actin and tubulin cytoskeletons are essential for induction of fiber bundling.

We continued to check the role of cellular actomyosin contraction, which is mainly regulated by RhoA-ROCK (Rho-associated kinase)-Myosin II signaling pathway [53]. In contrast to the control group, the fiber bundling disappeared after inhibition of ROCK with Y27632 (Fig. 5E). When inhibited actomyosin contraction with myosin adenosine triphosphatase inhibitor blebbistatin, or myosin light-chain kinase inhibitor ML-7, fiber bundling was also blocked (Fig. 5F and Fig. S6E and H). The images with more details are presented in Fig. S6A to E. Upon inhibition of small guanosine triphosphatase Rac1 with NSC23766 (40 μM), fiber assembly showed up with partial reductions, and cell migrations were less active (Fig. 5G and Fig. S6F). Similar results were observed with more reduced cell migrations at higher concentration of NSC23766 (100 μM) (Fig. S6G). The comparisons from fluorescent quantifications showed blocked fiber assembly after the contraction inhibitions, while with partial inhibition by Rac1 inhibitor (Fig. 5H). After the cytoskeleton or contraction inhibition, cell migrations were substantially reduced or shown in a scattering way (Fig. 5I and Figs. S5 and S6). These data together indicate that actomyosin contractions from cell clusters are essential for induction of COL fiber assembly.

### Calcium channels mediate cell clusters-induced fiber bundling

**Fig. 6.**
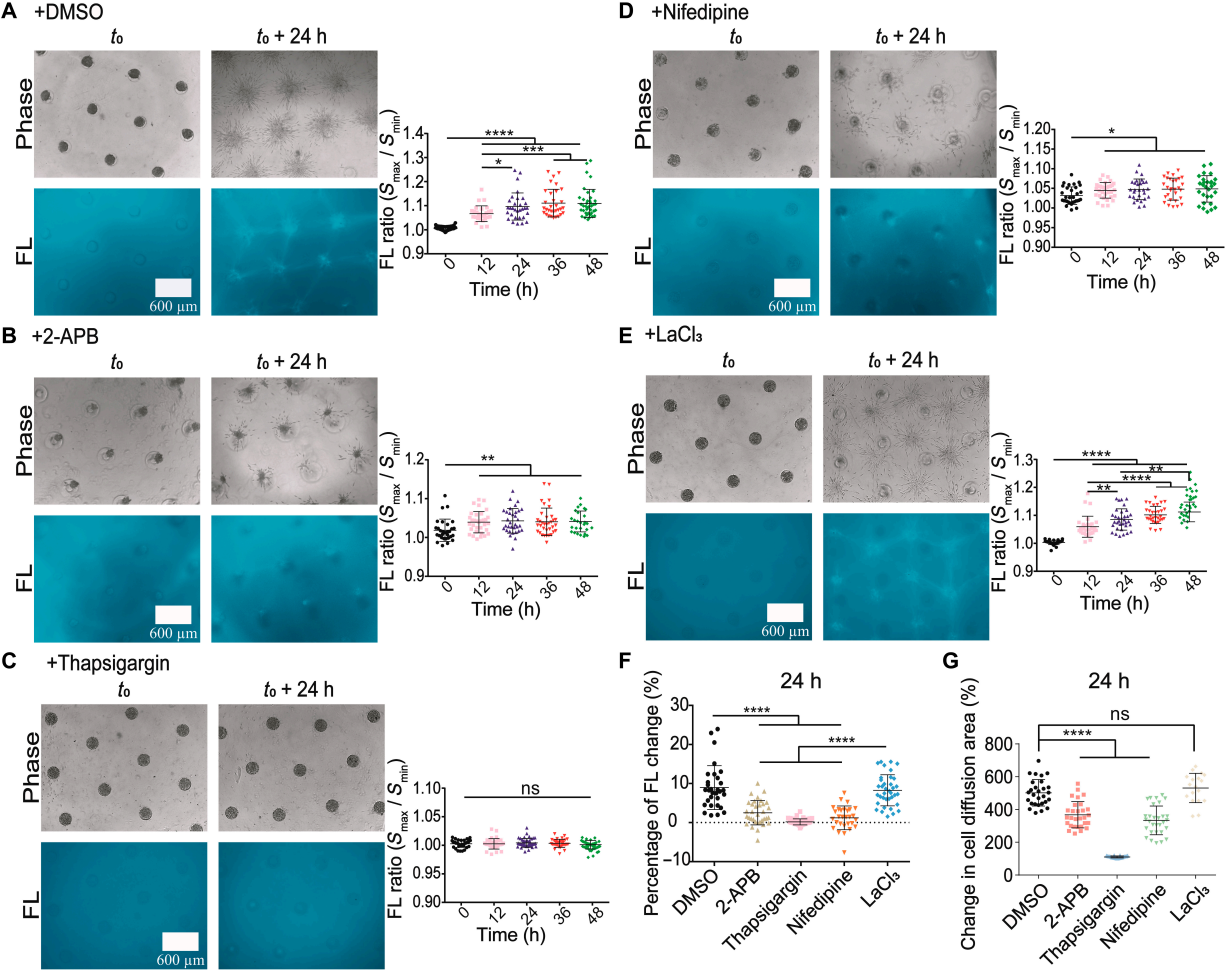
Calcium channels in regulating cell clusters-induced fiber assembly. Cell clusters were positioned into geometrical parallelogram arrays and treated with calcium channel inhibitors. (A to E) Fiber assembly and fluorescence quantifications at control condition (DMSO) (A) or treated with 20 μM 2-APB (IP_3_R channel inhibitor) (B), 10 μM thapasigargin (SERCA pump inhibitor) (C), 20 μM nifedipine (L-type channel inhibitor) (D), or 100 μM LaCl_3_ (SOC inhibitor) (E). (F and G) Comparison for fiber fluorescence changes (%) (F) or cell spreading areas from the clusters (G) in 24 h under the indicated conditions.

Our recent work showed that calcium channels at ER membrane are critical in cell–cell mechanical communications [27]; hence, the importance in the distant induction of fiber assembly was examined here. Similarly, COL fibers were assembled into network structure along with active cell migrations under control condition in the parallelogram pattern (Fig. 6A). When inhibition of inositol 1,4,5-trisphosphate receptor (IP_3_R) calcium channel or sarcoendoplasmic reticulum calcium ATPase (SERCA) pump at ER membrane with 2-APB or thapsigargin, both fiber assembly and cell migrations were almost blocked (Fig. 6B and C). Inhibition of L-type calcium channel at plasma membrane with Nifedipine also resulted into large inhibitions (Fig. 6D), whereas inhibition of membrane store-operated calcium channels (SOCs) with LaCl_3_ had little effect (Fig. 6E). The images with more details are shown in Fig. S7A to E. The percentage increases in fiber fluorescence quantification confirmed these observations (Fig. 6F). Cell migration quantification indicates marked inhibitions of cells spreading out from the clusters under additions of these calcium channel inhibitors except LaCl_3_ treatment (Fig. 6G). These results indicate that calcium signals regulated by the ER store and plasma membrane are important for cell-clusters-induced fiber assembly.

### The importance of membrane mechanosensitive integrin β1 and Piezo in remote induction of fiber bundling

**Fig. 7.**
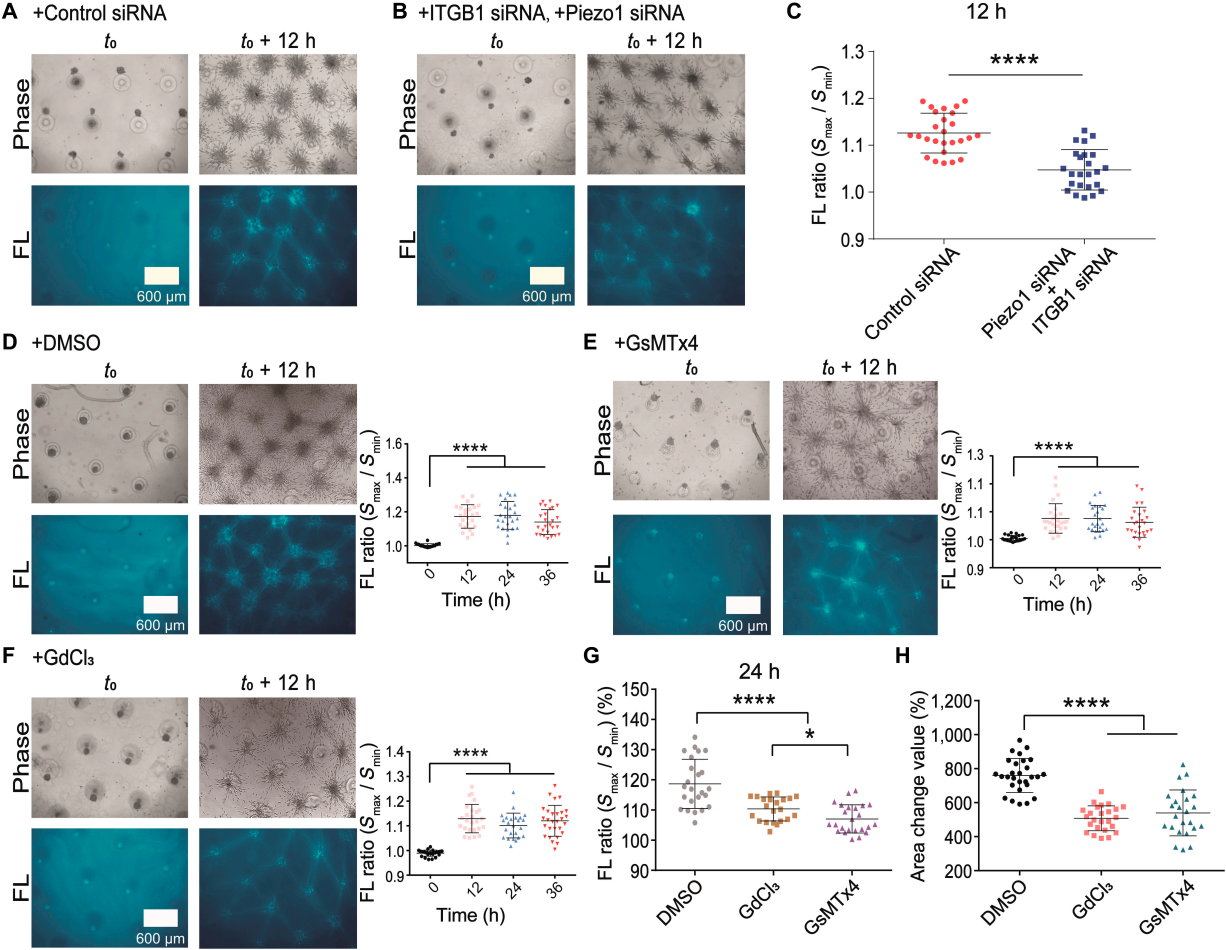
The role of integrin β1 and Piezo in fiber inductions. ASM cells were transfected with negative siRNA (100 nM) or double-positive siRNA for integrin β1 (ITGB1, 50 nM) and Piezo1 (50 nM). COL fiber inductions were recorded and quantified in geometrical parallelogram-arrayed clusters. (A to C) Fluorescent COL fibers and quantifications under the condition of cell clusters transfected with negative siRNA or double ITGB1 and Piezo1 siRNA. (D to F) Fluorescent COL fiber induction and quantification under the control condition (D), or with treatment of 5 μM Piezo inhibitor GsMTx4 (E) or 100 μM GdCl_3_ (F). (G and H) Comparisons of the percentage changes in fiber growth (G) or cell spreading areas from the clusters (H) in control and Piezo inhibitor groups.

Plasma membrane integrin and Piezo1 are mechanosensitive components [25,27]. Here, we examined their roles in the remote fiber induction. After partial down-regulation of integrin β1 expression with small interfering RNA (siRNA) transfection in airway smooth muscle (ASM) cells, COL fibers were partially induced and cell migration was less active in comparison to the control groups (Fig. S8A to C). Fluorescence quantification showed certain inhibition of fiber bundling with partial integrin β1 down-regulation (Fig. S8D and E). Then, we further examined double down-regulations of integrin β1 and Piezo1 with siRNA transfections in which COL fiber inductions were inhibited in comparison to the control group (Fig. 7A to C and Fig. S9A and B). These data indicate a possible coordinative role by integrin β1 and Piezo1 in the remote fiber induction, which is also supported by cell mechanosensing mechanism in recent work [54].

In considering that lung tissue expresses both Piezo1 and Piezo2 [55], we further tested the chemical inhibitors of Piezo 1 and 2 during the fiber induction by ASM cells. Two Piezo inhibitors GsMTx4 and GdCl_3_ both reduced the COL fiber assembly in the large-spatial parallelogram cluster arrays as well as the cell migrations (Fig. 7D to H). More details in images are shown in Fig. S10A to C. The data indicate that the 2 types of membrane mechanosensitive molecules integrin and Piezo together were critical in remote induction of fiber bundling by ASM cell clusters.

## Discussion

The long-range or large-spatial mechanical communications and resulted ECM remodeling provide new angle in understanding physiological homeostasis in vivo, tissue morphogenesis, and the progressing in certain diseases. In this work, we tried to investigate the biophysical mechanism of large-spatial fiber assembly induced remotely by cell mechanics and the supporting cell molecular mechanism. By incorporating dynamic contractions, MD along with FE modeling was able to simulate large-scale anisotropic COL fiber clustering under various geometrical distributions of cells. Experimental outcomes from cell clusters in spatial arrays yielded similar results to the simulated ones without spatial limitation, which also provided a platform in examining involved mechanical signals.

### Experimental system for cell active contraction-induced COL fibril bundling and large-spatial and dynamic simulations by MD and FE modeling

We employed micropatterns of cell cluster with various geometries to study cell active contraction-induced remodeling of COL fibril matrix (Fig. 1). The designed patterns provide a robust platform in examining the underlying mechanical mechanism with modeling simulations and cellular mechanosignaling studies.

According to our previous observations that cells-derived traction force was dynamic but not static in the hydrogels, MD simulation containing the dynamic factor was applied to model the COL fiber bundling induced remotely by cells (Fig. 2A to C). The simulated results were consistent with the calibrated maximum shear stress distributions from FE modeling (Fig. 2D to I), which did not have preconditional limitations like cell shape, cell number, geometry, or space. To check whether the simulated results occurred in reality, we designed experiments with various geometrical patterns for cell cluster distributions (Figs. 2J and 3). The geometrically arranged cell clusters including large-spatial square, parallelogram, and random-style patterns were covered into a layer of fluorescent COL hydrogel. The 3 types of arrays yielded highly consistent results with the MD- and FEM-simulated ones under similar setups (Fig. 3). These data largely verified the simulations based on cell dynamic shear tractions and also demonstrated the robustness of ECM remodeling from predesigns.

### COL fiber inductions in single polygons and their contraction-associated structural stability

Beside the large-scale arrays, it is also interesting to examine fiber inductions in single polygons with different geometries. The appropriately seeded cell clusters produced triangles, squares, parallelograms, pentagons, and hexagons through COL fiber remodeling (Fig. 4A to E and Fig. S4). Due to the assembled fiber scaffolds induced by cell contractions, their structural stability is measured in area changes during 48-h culture. The large square array (~20% shrinkage) is more stable than parallelogram and random-style ones (~40% to 45% shrinking) (Fig. 4F, left graph). In single polygons, the squares are the most stable (~18% shrinking), followed by pentagons and hexagons (~25% shrinking), then parallelograms (~30% shrinking) and triangles (~45% shrinking) (Fig. 4F, right graph). The stability of the assembled scaffolds can reflect the internal contraction strength and balance.

This helps explain cell contractions-induced COL remodeling and implicates the existence of tensions in maintaining stability of the fiber structures. It is consistent with recent report that the fibers bear the major tensions between cells [41]. The MD simulations predicted the fiber clustering in single parallelogram (Fig. 4G), but not yet tension-mediated structural stabilities.

### The cellular mechanical mechanism in mediating the remote inductions of fiber bundling

It becomes evident that cell mechanics induces the large-scale COL fiber bundling, and the mechanical communications between cells promote cell directional migrations. The intracellular mechanical signals were further investigated in understanding the associated mechanism. Cells are both contraction force generators and force sensors in this experimental system. The roles of different cellular mechanical components were studied, such as force generation (actomyosin), force transductions (calcium signals, cytoskeletons), and force mechanosensors (integrins, Piezo).

Inhibitions of cytoskeletons (actin microfilaments and microtubules) or myosin contractility blocked the fiber inductions (Fig. 5), which demonstrates cell contractions-dependent remodeling of COL hydrogel. It is noted that tubulin cytoskeleton, which does not provide cellular contraction force but gives mechanical supports, is also essential for the remote fiber inductions (Fig. 5C and D). Although there is no direct data to interpret this result yet, it may be related to microtubule functions in motility, intracellular transportations, and mechanics [56,57].

Calcium is mechanosensitive signal in cells [57,58]. In this work, inhibitions of ER calcium channels IP_3_R and SERCA pump, or plasma membrane L-type calcium channel largely reduced the capability of cell clusters in distant induction of fiber assembly (Fig. 6). Therefore, the physiological flow of Ca^2+^ between cells and the microenvironment or between the cytoplasm and ER store is essential in driving the hydrogel remodeling. Besides other rich functions of calcium signals, in regarding the role of Ca^2+^ in regulating actomyosin contractility [59,60], it can make connection for mechanotransduction of these calcium channels in the fiber inductions.

Studies have shown the importance for the membrane mechanosensory integrin (β1) or Piezo1 in cell distant mechanical interactions [25–27]. Here, we further examined the role in cell distant induction of fiber assembly. By applications of siRNA-transfection method or chemical inhibitors, integrin β1 and Piezo1showed important roles in mediating the fiber bundling (Fig. 7A to G). Since the remote fiber bundling is induced from active contraction of cell clusters, integrin may have the role in mechanical transmission as well between cells and the surrounding COL matrix.

Although cell migrations were not a major focus in this work, the cytoskeletons of microfilaments and microtubules seemed essential for cell movements in the experimental model (Fig. 5A to D), and myosin contraction, ER calcium channels, and Piezo are critical in cell directional migration between clusters (Figs. 5F to I, 6, and 7).

Together, these mechanobiological components are indispensable with different functions in cell remote mechanics-induced fiber bundling as well as directional cell migration. Integrins and Piezo are located at the plasma membrane and play the roles as mechanical force transmission and sensors. Piezo activation induces intracellular calcium increase, which can further trigger downstream Ca^+2^-dependent signal pathways [61]. ER calcium channels are sensitive to external stress and transduce the mechanical signals in cells such as activation of myosin. Actomyosin generates contraction force to drive the fiber bundling and cell migration.

### The prospective application for cultured scaffolds in tissue engineering

This work also yields one principal experience: to culture cell biomechanics-induced scaffolds or tissue patterns according to predesigns. Tissues and organs have specific shapes or heterogeneous structures, which has been one intriguing topic in developmental biology but also a challenge in tissue engineering. Here, based on inductions of COL matrix remodeling with predesigned cluster distributions, we successfully produced square/parallelogram/random-array lattices with assembled fibers (Fig. 3) and also single polygonal structures from triangles to hexagons (Fig. 4), although their structural stabilities were variable. This may lead to improved strategy for microtissue bioengineering including neural network, skin tissue, and microvascular system with this supplemental recipe in the current field.

## Conclusions

**Fig. 8.**
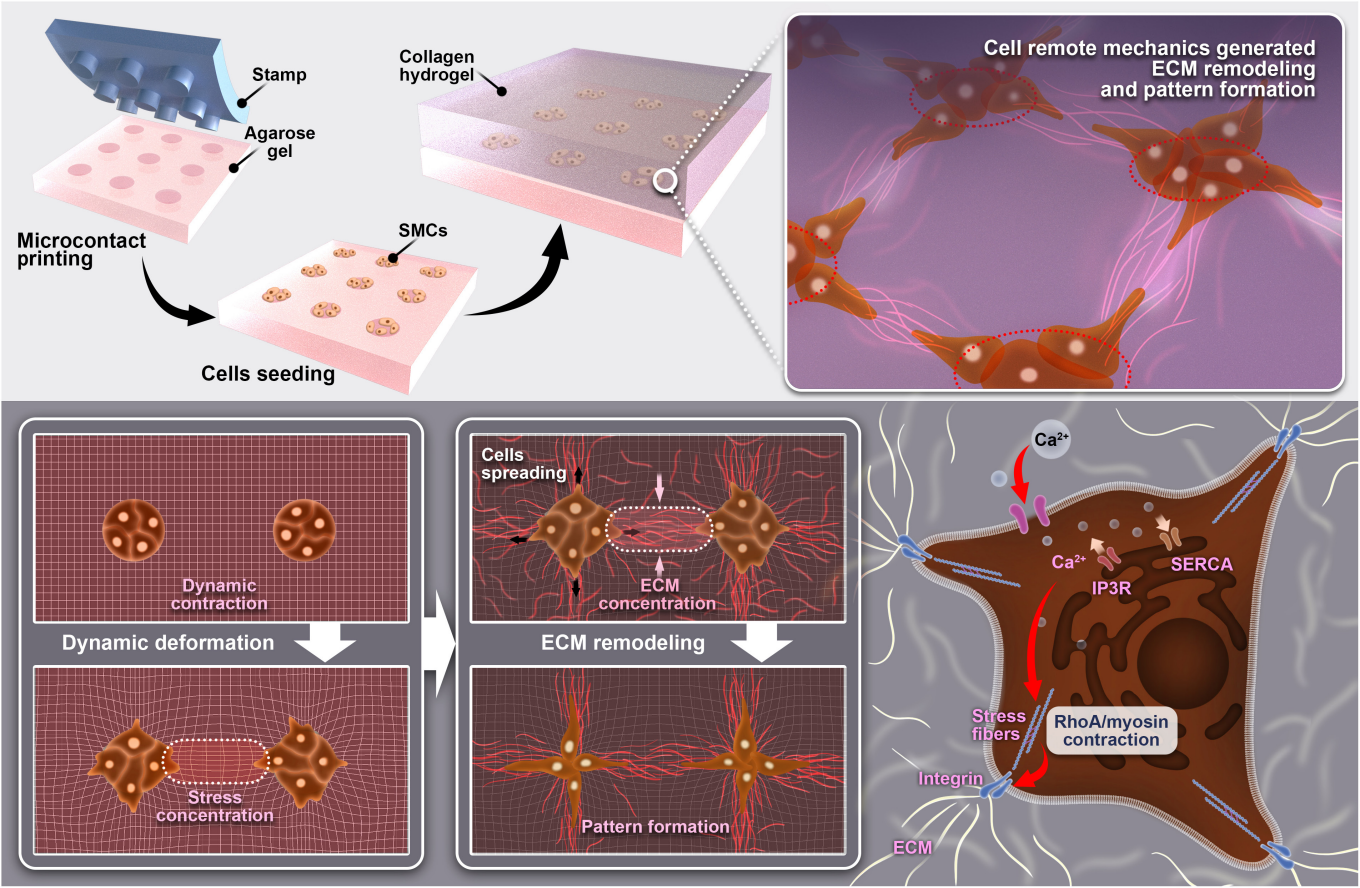
Illustration for distant induction of collagen hydrogel modeling by cell dynamic mechanics. MD simulations show anisotropic stress concentrations and collagen fiber clustering by cell mechanics, along with associated cellular biomechanical mechanism, which implicates the heterogeneous organization of tissues maybe partially originated from the anisotropic distribution of cell mechanics. (Detailed descriptions seen in the text of Discussion.)

This work provided new mechanistic insights into dynamic and spatial factors on the anisotropic induction of ECM remodeling by cell active contraction at a large scale, as well as the associated cellular mechanical mechanism. As depicted in Fig. 8: by incorporated dynamic contractions, the simulations with MD and FE modeling yielded highly matching outcomes without spatial limitation to spatially anisotropic COL fiber bundling in experiments from large square, parallelogram, and random-style patterns of cell cluster cultures. The designed single polygons with various geometries led to well-predicted structures with assembled COL fibers. The cell cytoskeletal integrity (actin filaments and microtubules), actomyosin contractions, and ER calcium channels acting as force generations and transductions were essential for distant induction of COL fibers, while membrane mechanosensory components integrin β1 and Piezo1 show important role in regulating the fiber assembly. The assembled scaffolds based on predesigns may also lead to application in microtissue engineering. The conclusion implicated that the heterogeneous structures of tissues and organs might be partially derived from the anisotropic nature of cell mechanics.

## Materials and Methods

### Cell culture and siRNA transfection

Rat ASM cells were isolated from the tracheal tissue of 6- to 8-week-old female Sprague Dawley rats as described previously [62]. The cells were cultured in low-sugar Dulbecco’s modified Eagle medium (Invitrogen) supplemented with 10% fetal bovine serum (GIBCO) and penicillin/streptomycin antibiotics (GIBCO). The cells used in the experiments were generally maintained within 10 passages.

To perform siRNA transfections, ASM cells were seeded into a 6-well plate at ~40% density, and the next day, rat integrin beta1 (ITGB1) siRNA (Thermo), Piezo1 siRNA (Horizon Discovery), or control siRNA (50 nM in 2-ml medium) was transfected into the cells by using 5 μl of Lipofectamine 3000 reagent. After incubation for about 8 h, the medium was changed, with a waiting period of 48 to 72 h before further experiments.

### Preparation of polydimethylsiloxane molds

The micropatterned arrays of cell clusters were prepared according to the method derived from our previous work [13], but with more variably geometrical distributions. Basically, we used the software AutoCAD to design the patterns of circle distributions, in which the circles were set as 200 μm in diameter, and depth of the pattern is 200 μm, and the distance between the centers of circle is 600 to 800 μm. The parameters for different geometrical patterns are listed in Table 1. The silicon wafers printed with the designed patterns were manufactured by Suzhou MicroFlu Technology Co. and In Situ Co., Ltd.

**Table 1.** The parameters for various geometric patterns

Distribution geometries of circles	Parallelogram arrays	Square arrays	Random arrays	Triangle	Parallelogram	Square	Pentagon	Hexagon	Triangle (+)	Parallelogram (+)	Square (+)	Pentagon (+)	Hexagon (+)
Geometric patterns	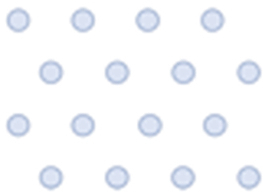	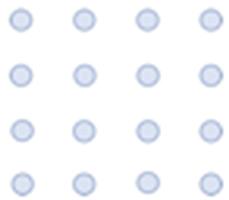	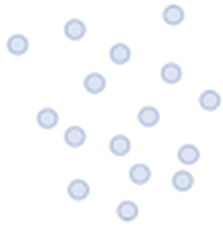	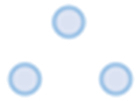	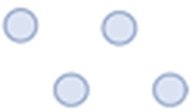	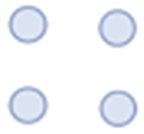	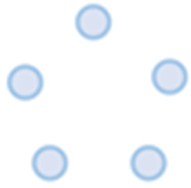	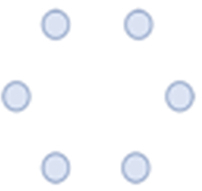	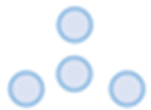	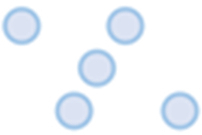	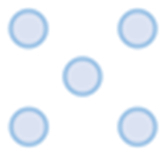	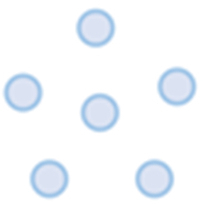	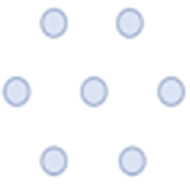
Mutual spaceration (μm)	800	800	/	600	600	600	600	600	600 (side)	600 (side)	600 (side)	600 (side)	600 (side)

To fabricate the polydimethylsiloxane (PDMS) molds, the 2 liquid components from the Sylgard 184 Kit (Dow Corning) were mixed at a mass ratio of 10:1 (base to catalyst 87-RC) and poured onto the silicon wafer after extraction of air bubbles by vacuum, then cured at 80 °C for over 4 h, and left at room temperature overnight. The solidified PDMS was carefully peeled off from the wafer and further cut into small pieces (~ 1 × 1 cm) to adapt the next experimental utility. The prepared PDMS molds were soaked in 75% ethanol for disinfection and exposed to ultraviolet light for several hours in the cell culture hood before each experiment.

### Expression and purification of EGFP-CNA35 protein

The pET28a-EGFP-CNA35 DNA construct purchased from Addgene was described before [63]. The procedures for expression and purification of EGFP-CNA35 protein were introduced in our recent work [20]. In brief, the DNA plasmid was transformed into BL21 (DE3) competent bacteria, and when culturing in the lysogeny broth medium, the protein expression was induced by isopropyl β-D-1-thiogalactopyranoside at room temperature. The precipitated bacteria were lysed with B-PER lysate solution (Thermo Scientific), and HisPur Ni-NTA agarose beads were added to pull down the 6xHis-tagged protein. The concentration of purified protein was measured by Branford protein assay kit (Thermo), and protein aliquots were stored at −80 °C.

### PDMS mold printing onto agarose gel and cell cluster culture

First, to prepare cell clusters on micropatterned agarose gel, agarose solution (20 g/l) was made by dissolving 2 g of agarose (Thermo) in 100 ml of sterile phosphate-buffered saline (PBS) by microwaving, and ~150 μl of agarose solution was dropped onto the glass-bottom dish. The sterilized PDMS mold was placed onto the solution and slightly pressed downward. After solidification at room temperature, the mold was carefully peeled off without disruption of the printed patterns on agarose gel. Suspension of ASM cells in medium (200 μl; ~1.2 × 10^6^ cells/ml) was added onto the agarose gel, and after 10 to 15 min of waiting for cells to settle down into the pattern areas, the additional medium was absorbed away and the patterned regions were washed once or twice with culture medium carefully. Then, cells were generally filled into the circular caves (200 μm in depth) without much left on other areas (cells are nonadhesive on agarose gel).

Second, to cover the cell clusters with fluorescent COL (Cultrex 3-D Culture Matrix Rat Collagen I) hydrogel. The preparation of COL solution was carried out on ice: completely mixed the rat tail COL solution (4 mg/ml) with purified EGFP-CNA35 protein (diluted to 1 mg/ml in advance) at the volume ratio of 5:1, and waited for about 15 min; afterwards, added neutralizing solution at 1:9 volume ratio of the COL one, and further diluted the COL to final 1 mg/ml (or other alternative concentration) with PBS. Then, the mixed COL solution (300 to 400 μl) was added onto the patterned cells-containing agarose gel, and we tried to spread the COL liquid across the glass-bottom surface of the dish in addressing nonadhesive COL gel onto agarose. Then, the assembled cell samples were placed in the culture incubator at 37 °C for 15 to 20 min, and after gel formation, 2 ml of regular culture medium was added into the dish. In this way, the cell clusters were generally embedded into COL while underneath the gel layer.

### Cell cluster cultures along with inhibitor applications

Before detached for generating pattern of cell clusters, ASM cells cultured in 6-well plates (NEST) were preincubated with the inhibitors for 1 to 2 h, and the solvent DMSO (Beyotime) was used as control. After cell clusters were sandwiched between COL and agarose gels, the culture medium containing the same inhibitor was added back. To enhance the medium diffusion with inhibitor through the hydrogel, a small hole was created at the edge of the gel with a fine needle without disturbing the cell mass. After culture for 24 hours, 1 ml of more medium with the same inhibitor was further supplemented to the culture in considering the inhibitor stability at 37 °C. The formation of COL fiber bundles and cell migration were recorded under microscopy.

The applied inhibitors in the experiments were mostly purchased from Sigma, including 2-Amino-ethoxydiphenyl borate (2-APB, an IP_3_R calcium channel inhibitor with cell permeability, 20 μM), nifedipine (membrane L-type calcium channel inhibitor, 20 μM), Lanthanoum(III) chloride (LaCl_3_, SOC calcium channel inhibitor, 100 μM), nocodazole (microtubule de-polymerizing agent, 1 μM), blebbistatin (myosin II adenosine triphosphatase inhibitor, 40 μM), CytoD (actin polymerization inhibitor, 1 μM), ML-7 (MLCK inhibitor, 40 μM), NSC23766 (Rac1 activation inhibitor, 100 μM), and LatA (microfilament polymerization inhibitor, 237 nM). Y27632 (ROCK inhibitor, 40 μM) was from MedChemExpress, and thapsigargin (ER SERCA pump inhibitor, 10 μM) from Abcam.

### Quantitative polymerase chain reaction measurements

After ITGB1 or Piezo1 siRNA transfection, the decrease of mRNA expression in ASM cells was evaluated by real-time quantitative PCR (qPCR), of which the details were described in our recent work [64]. The primer sequences of qPCR derived from others’ studies are listed on Table 2 [65,66].

**Table 2.** The primer sequences of qPCR for measuring ITGB1 and Piezo1 mRNA levels

Gene name	Primer (forward)	Primer (reverse)	Company
ITGB1 [66]	GAATGGAGTGAATGGGACAGGAG	CAGATGAACTGAAGGACCACCTC	GENERAL BIOL
Piezo1 [67]	GACGCCTCACAAGGAAAGC	GGGCAGCATCTATGTCATCC	
GAPDH	AGGTCGGTGTGAACGGATTTG	GGGGTCGTTGATGGCAACA	

### MD simulations of fibers clustering and reconstruction

A 2D network model based on MD simulation was introduced to investigate the mechanics of fibers cluster and cell migrations. In the simulations, the fibers are modeled by series of spring bonds that can be stretched and compressed. The collagen fibers were further simplified as 2D fibers, and their cross-links were modeled as the jointed nodes between/among different fibers. The collagen fiber is considered as an anisotropic elastic material that has a high modulus in tension and a very low modulus in compression [50]. The bond’s tension stiffness was set as 100 times of compression stiffness. The fibers’ cross-links may reconstruct under tension or compression. Here, we set a criterion for the reconstruction that is the bond length change being higher than 50% of bond length. The cross-link of this fiber will be reconstructed, and this bond length will change from its initial value to its current value. The cluster of cells was shown as a circle with 83 nodes. The Langevin dynamics were performed by using GROMACS package [67]. The XYZ coordinates of cells nodes and Z coordinates of cross-link nodes were fixed during simulation. The dynamic contraction of cells was applied by reducing the size of cell cluster to simulate the predeformation of cells. The radius of cluster was reduced by 5% per 50,000 simulation steps. The simulation results were analyzed with VMD program [68].

### FEM for stress distributions on the hydrogel

The FEM model for analyzing the relationship between matrix stress distribution and the fibers network formation has been introduced in our previous studies [20,69]. The FEM is a powerful technique in solid mechanics and computational mechanics. It involves dividing a complex structure or system into smaller, simpler elements, allowing us to approximate its behavior. By solving equations for each element and combining their effects, we can understand how the overall system responds to various forces and conditions. Generally, the governing equations of the system in FE method include equilibrium equation, constitutive equation, and boundary condition. Briefly, the interface of cells and collagen matrix was modeled as isotropic linear elastic plane surface. The boundary of plane surface was fixed. The cell region was removed. The cell traction force was applied on the edges of cells’ region to mimic the contraction force of cells on the matrix. The stress value on the matrix was normalized for a quantitative analysis. The FEM model was solved and visualized by ABAQUS packages.

### Live cell imaging, quantifications of COL fiber intensities, and cell spreading areas

The live cell microscopy workstation (Zeiss) was equipped with an X-Y-Z controller system for multiposition imaging, fine autofocusing function, and a culture chamber loaded on the sample stage to maintain 5% CO_2_ and the temperature at 37 °C. Most fluorescence imaging experiments were carried out with ×5 and ×10 objectives under the microscope. One round of the experiments spanned 2 to 3 d, and the images for fluorescent collagen fibers (fluorescein isothiocyanate channel) and cell migrations (differential interference contrast channel) were taken at different time points as indicated, for example, the initial status, 12, 24, 36, and 48 h.

ImageJ was used to quantify the relative fluorescence intensities (FL) of collagen fiber bundles in contrast to the close non-fiber areas, as demonstrated in Fig. S1. In the procedures, the image was rotated so the COL fibers were displayed in horizontal direction; a constant rectangular region (ROI) was chosen while the fiber bundle was positioned at the middle of ROI. Averaged FL was measured from left toward right along the selected rectangular ROI by using the “plot profile” function in ImageJ. The acquired values from each ROI were input into the Excel file, which generated an averaged fluorescence distribution curve along the selected region. Then, the sum (*S*_*max*_) of 7 points around the maximum intensity value (3 values from the left and another 3 from the right) was calculated, as well as the sum (*S*_*min*_) of 7 values from 2 ends of the curve (3 or 4 values from each) in representing the low nonfiber intensities. The FL ratio was defined as *S*_*max*_ divided by *S*_*min*_ (Ratio = *S*_*max*_/S_*min*_). Generally, the COL fluorescence images were quantified from 4 or 5 given time points within 48 h, for instance, 0, 12, 24, 36, and 48 h. Statistical comparison was conducted between the initial time point and later ones, which illustrates the formation of COL fibers induced by cell clusters remotely.

Cell spreading areas in the clusters were also measured using ImageJ based on the bright-field microscopic images. The cells migrating out of each cluster were tracked as many as possible at the boundaries to generate a circled region around the cluster at the given time points. The spreading areas of cells from the tracked regions were then obtained by ImageJ. The percentage change of each cell cluster area was calculated based on cell spreading from the original patterned cluster size after 24-h culture. The calibrated change in cell spreading area did not subtract the original cluster size. Then, GraphPad Prism 6.0 was used to analyze and compare the differences in cell migration ability under different experimental conditions.

The COL fluorescence curves and graphs of scattering dots from fluorescence ratio or cell migrations were processed by Origin 2020, GraphPad Prism 6, and Excel software. Student *t* test was applied for statistical analysis. *, **, ***, **** represent “*P* value” < 0.05, 0.01, 0.001, 0.0001 for significant difference, respectively, while “NS” for no significant difference.

## Data Availability

Where all data needed to evaluate the conclusions of the study are available (such as present in the paper and/or the Supplementary Materials).
