# Supplementary material for: Cell Mechanics Regulates the Dynamic Anisotropic Remodeling of Fibril Matrix at Large Scale
Source: Research (Wash D C). 2023 Nov 15;6:0270. doi: 10.34133/research.0270 (PMC11776286; doi:10.34133/research.0270)
Supplement: Supplementary 1 — Figs. S1 to S10 [file research.0270.f1.pdf]

Supplementary information

Cell mechanics regulates the dynamic anisotropic remodeling of fibril matrix at large scale

Mingxing Ouyang[1,+,*],Yanling Hu[1,+], Weihui Chen[1], Hui Li[1], Yingbo Ji[1], Linshuo Qiu[1], Linlin Zhu[1], Baohua Ji[2], Bing Bu[1,*], Linhong Deng[1,*]

[1]Institute of Biomedical Engineering and Health Sciences, School of Medical and Health Engineering & School of Pharmacy, Changzhou University, Changzhou, 213164 China

[2]Institute of Biomechanics and Applications, Department of Engineering Mechanics, Zhejiang University, Hangzhou, 310027 China

[+]Shared first authorships; [*]Corresponding authors.

The supplementary information contains 1 movie and 10 figures.

Supplementary movie and figures

Movie 1: The dynamic process by Molecular Dynamics simulation shows the fiber emergences in connecting the cell clusters in random-style pattern in corresponding to the data of Figure 2I.

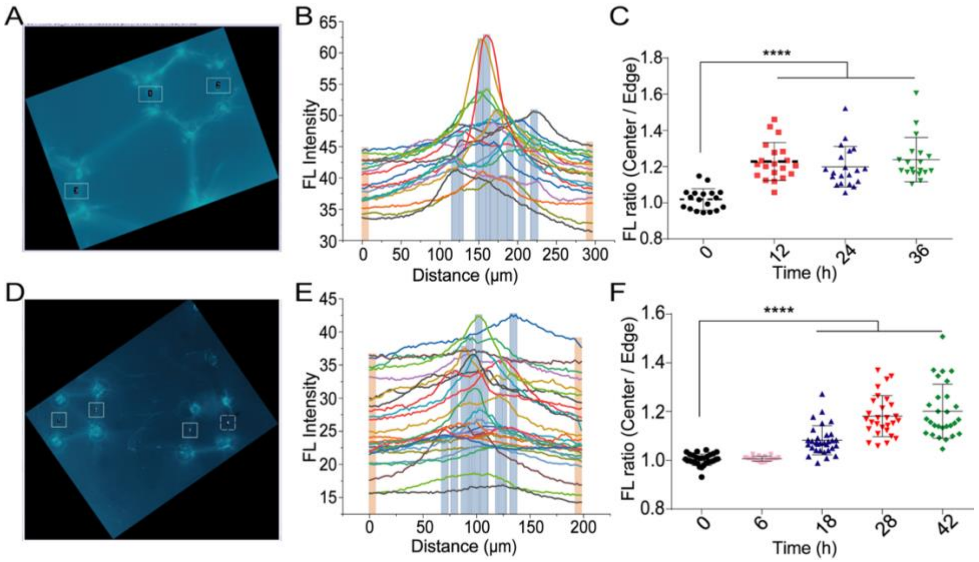

Figure S1. The procedural demo for COL fluorescence quantifications. The processing details with text were described in the Methods. (**A-C**) The sample of single hexagon for COL fluorescence quantifications. (A) the COL fluorescence image along with selected regions (ROI); (B) the fluorescence distributions of individual ROIs; (C) fluorescence-intensity (FL) ratio quantifications (mean ± S.E.M.) at the different time points. (**D-F**) The sample of single parallelogram for the fluorescence quantifications.

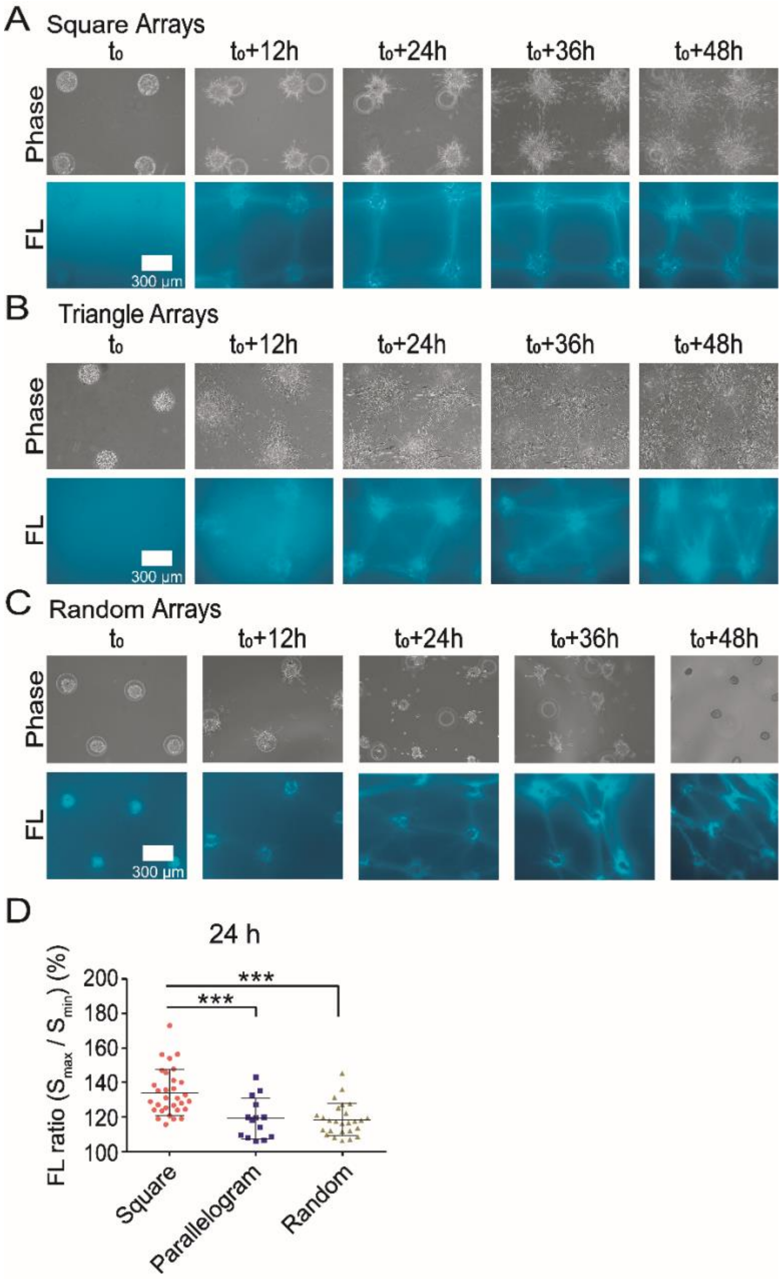

Figure S2. The COL fiber inductions along the culture times in different geometrical arrays. The images were taken with x10 objective, in corresponding to the images taken with x5 objective in Figure 2. **(A-C)** The representative bright-field and COL fluorescence images along the culture time in large-spatial square (A), parallelogram (indicated as 'Triangle Arrays' here) (B), and

random-style (C) arrays. **(D)** Comparisons of COL fiber bundle strengths among the three types of paterns based on quantified COL fluorescence ratio at 24 h.

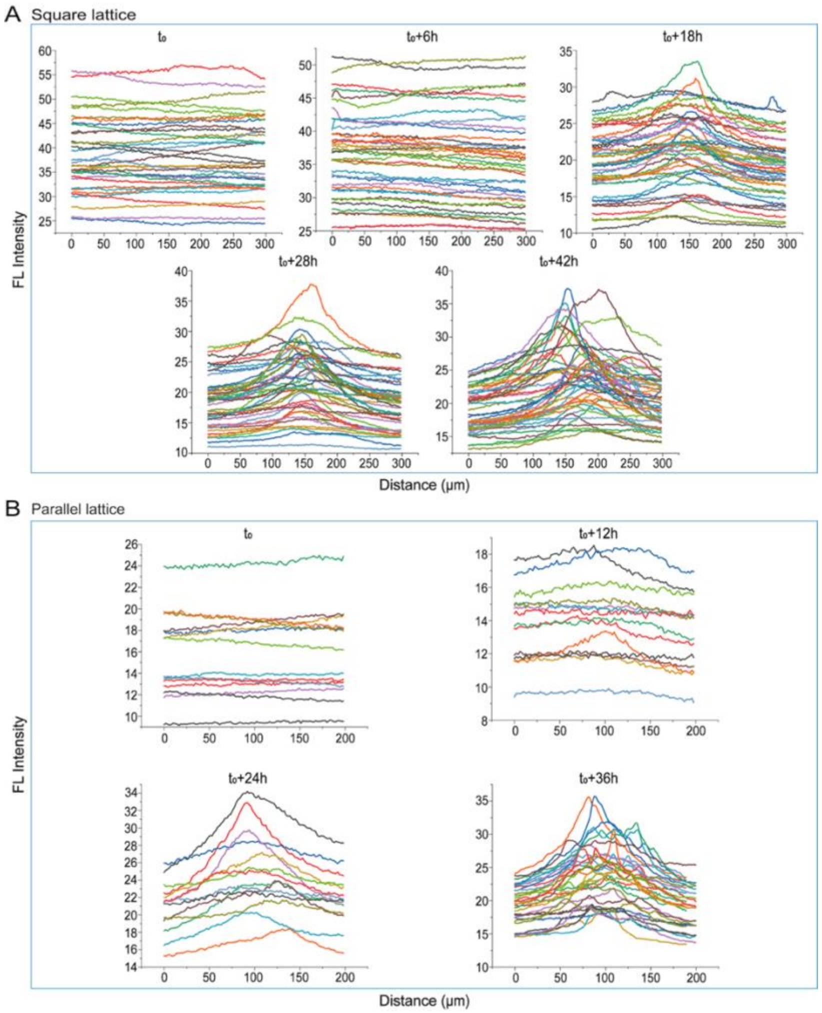

Figure S3. COL fluorescence distribution curves along the individual selected regions. As representative demonstrations, the quantifications of fluorescence (FL) curves showed the gradual emerging COL fibers at the different culture time. **(A, B)** The curves of COL fluorescence distributions at the large-spatial square pattern (A), and parallelogram pattern (B).

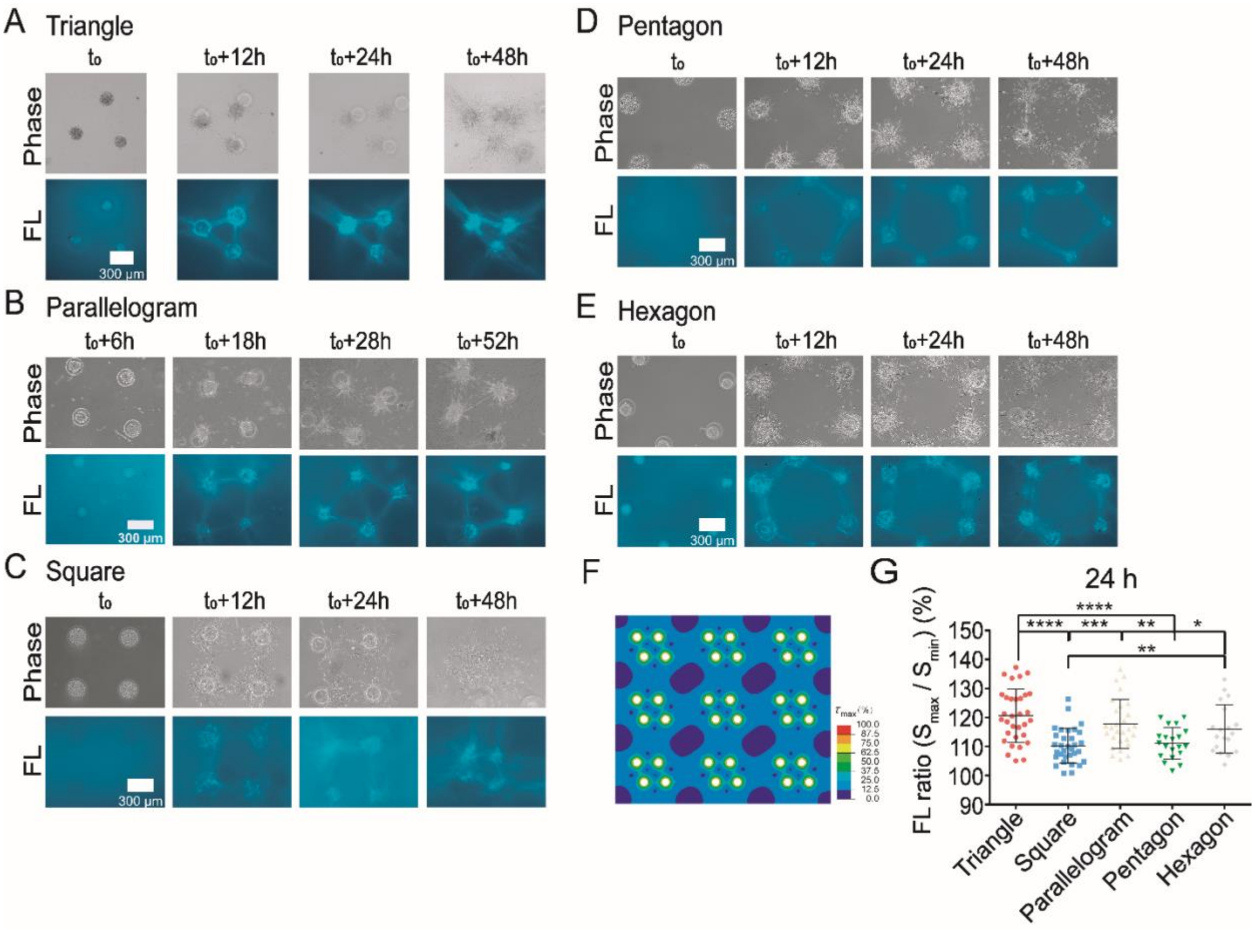

Figure S4. COL fiber inductions in single polygons with diverse geometries. The images were taken with x10 objective, in corresponding to Figure 3. **(A-E)** The representative bright-field and COL fluorescence images from single triangles to hexagons. **(F)** The maximum stress distribution in single parallelogram by FEM stimulation. **(G)** Comparisons of COL fiber bundle strengths among the different single polygons based on quantified COL fluorescence ratio at 24 h.

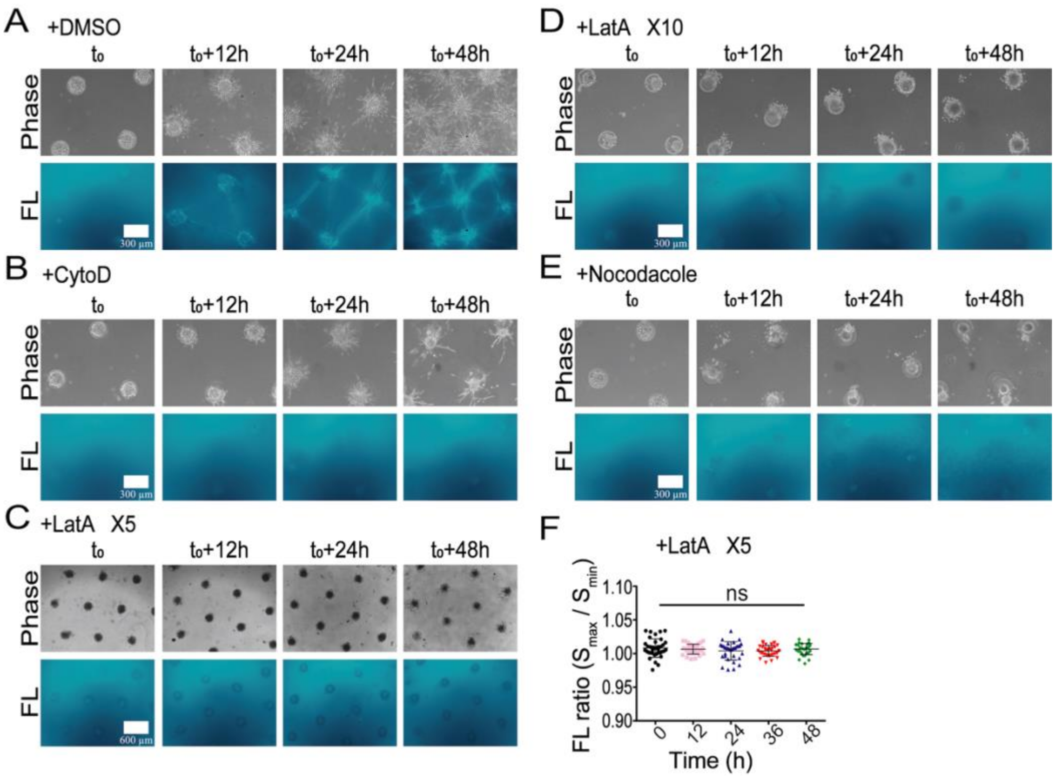

Figure S5. The importance of cellular cytoskeleton integrity in remote COL fiber inductions. The images were taken with x10 or x5 objective, in corresponding to Figure 4(A-D). **(A-F)** The representative images at the different culture time under control condition (DMSO) or treated with CytoD, LatA or Nocodazole (A-D), and fluorescence quantification for LatA treatment (F).

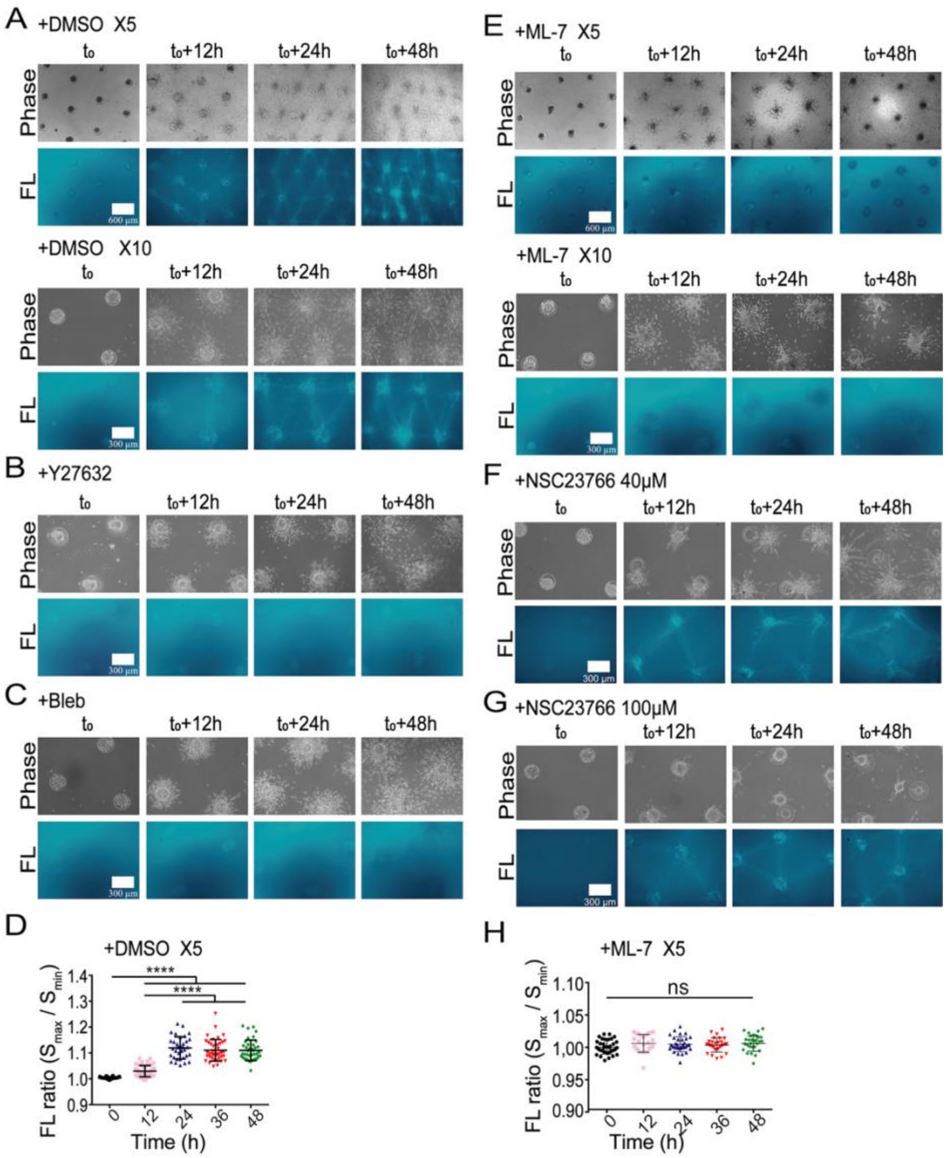

Figure S6. The importance of actomyosin contraction in remote COL fiber inductions. The images were taken with x10 or x5 objective, in corresponding to Figure 4(E-H). **(A-H)** The representative images at the different culture time under control condition (DMSO) or treated with Y27632, Blebbistanin, ML-7, or NSC23766 at 40 or 100 μM, and fluorescence quantification for DMSO (D) or ML-7 (H) treatment.

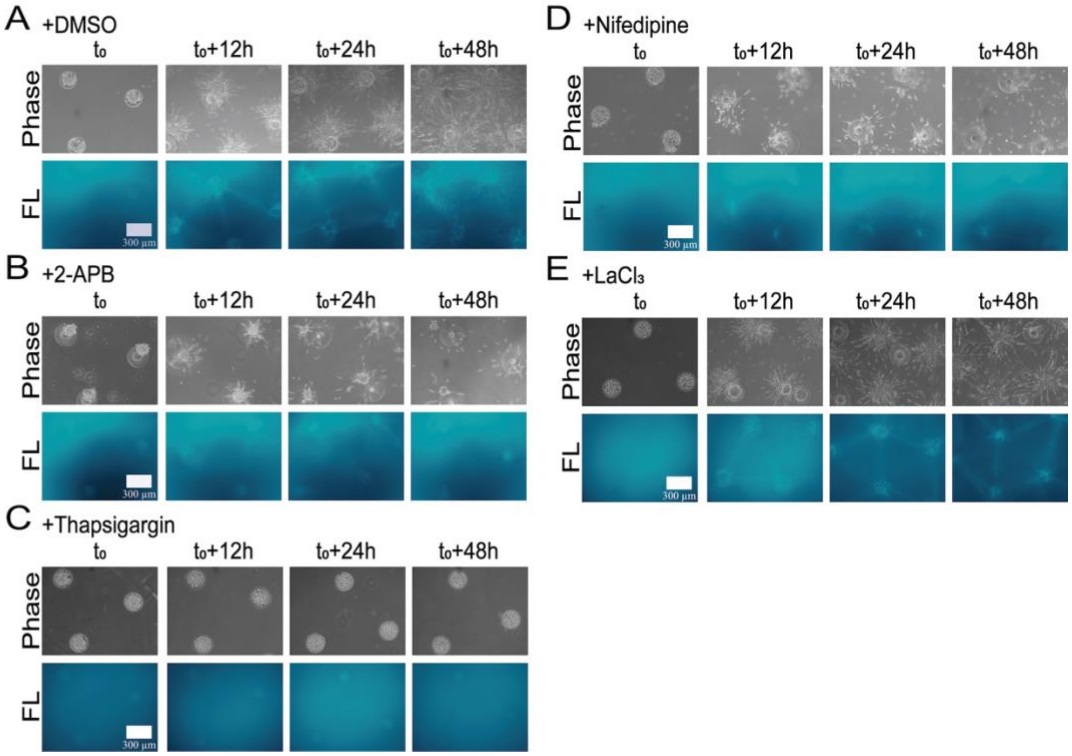

Figure S7. Measurements of COL fiber inductions under inhibitions of calcium channels. The images were taken with x10 objective, in corresponding to Figure 5. **(A-E)** The representative bright-field and COL fluorescence images under control (DMSO) or treated with 2-APB, Thapsigargin, Nifedipine, or $LaCl_3$.

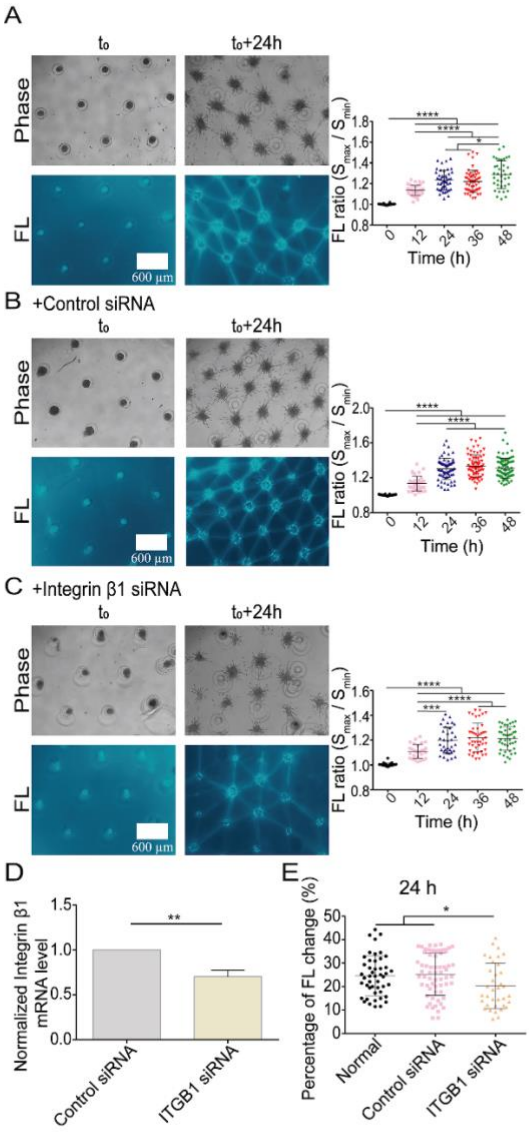

Figure S8. The remote COL fiber inductions under transfection with ITGB1 siRNA. ASM cells were transfected with positive or negative siRNA (50 nM) for integrin β1 (ITGB1) or Piezo1. COL fiber inductions were recorded and quantified in geometrical parallelogram-arrayed clusters. **(A-C)** Fluorescent COL fibers and quantifications under the condition of cell clusters without transfection (**A**), or with negative siRNA (**B**) or ITGB1 siRNA (**C**) transfection. **(D, E)** The mRNA expression levels (**D**) and comparison of percentage changes in fiber growth (**E**) with ITGB1 siRNA transfection.

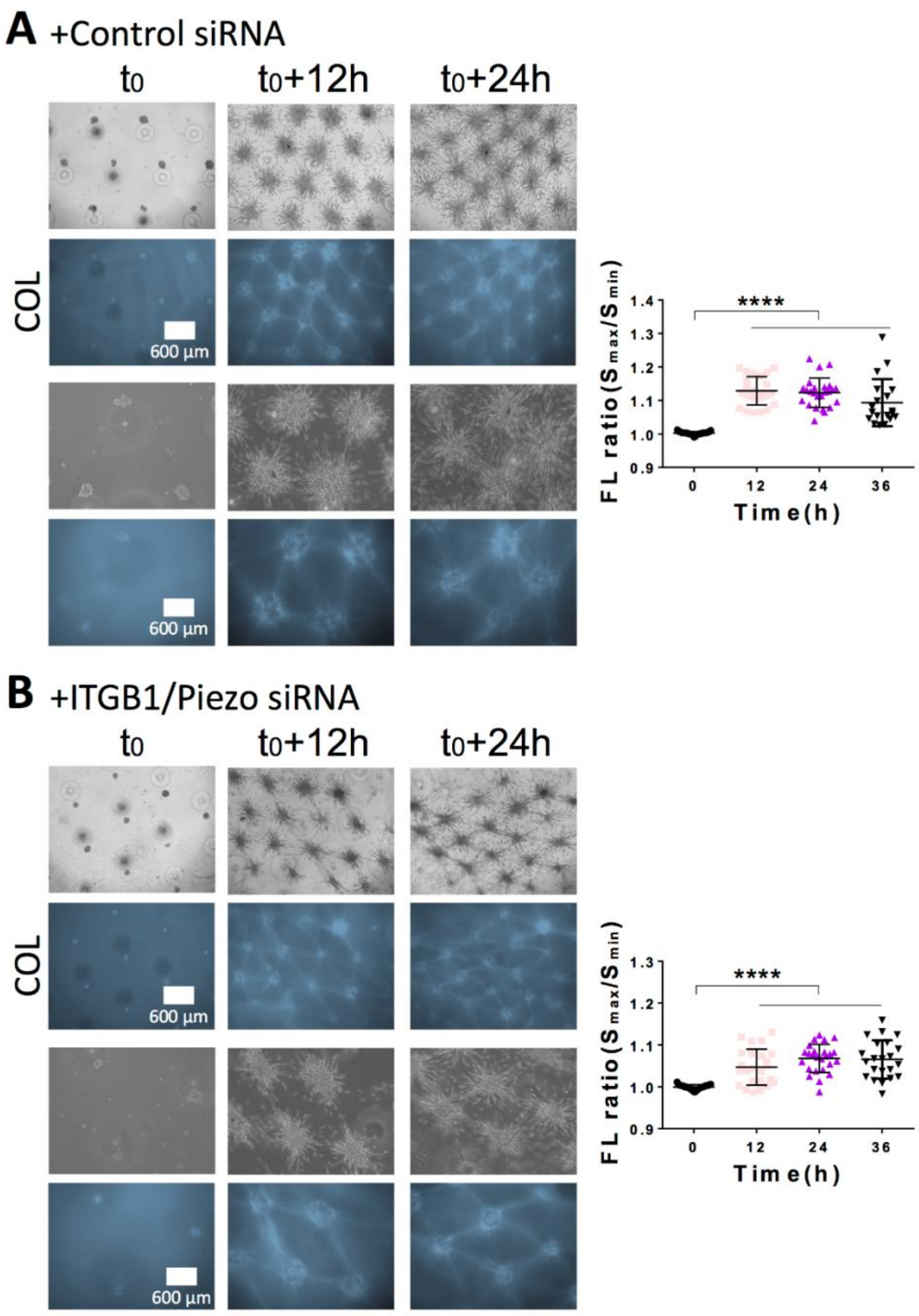

Figure S9. The role of double down-regulations of integrin β1 and Piezo1 by siRNA transfection in COL fiber inductions. The images were taken with x10 objective, in corresponding to images in Figure 6(A, B). **(A, B)** The representative bright-field and COL fluorescence images transfected with negative siRNA or double ITGB1 and Piezo1 siRNA.

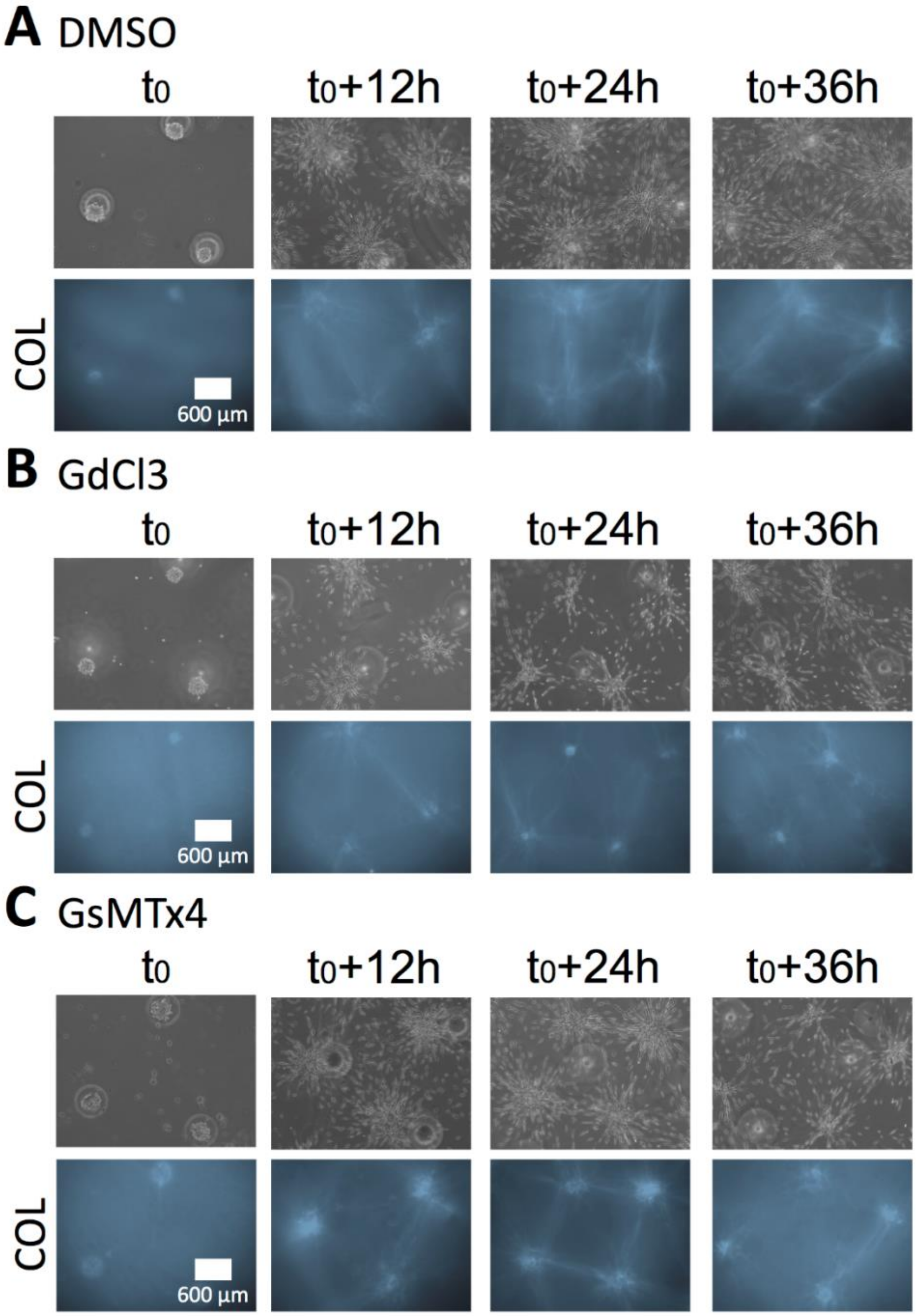

Figure S10. The role of Piezo inhibition in COL fiber inductions. The images were taken with x10 objective, in corresponding to images in Figure 6(D-F). **(A-C)** The representative bright-field and COL fluorescence images under control condition, or treated with GsMTx4 or $GdCl_3$.
